# Supplementary material for: Somatostatin-Expressing Neurons Regulate Sleep Deprivation and Recovery
Source: Genes (Basel). 2026 Jan 1;17(1):51. doi: 10.3390/genes17010051 (PMC12840664; doi:10.3390/genes17010051)
Supplement: Supplementary file 1 [file genes-17-00051-s001.zip › Supplementary File S5-Suuplementary_Tables.pdf]

Table alpha genome enrichr

Kobaysahi Kenta

September 2025

$\ell_1 = 2$

Table S1: left cerebral cortex up1

Index	Name	P-value	Adjusted p-value	Odds Ratio	Combined score
1	Mouse 96 Sst up	0.0004666	0.08772	2.38	18.26
2	Mouse 312 NP SUB up	0.001048	0.09848	16.60	113.92
3	Mouse 309 L5 NP CTX up	0.02963	0.55990	40.19	141.43
4	Mouse 318 CT SUB up	0.02963	0.55990	40.19	141.43
5	Mouse 314 NP SUB up	0.03931	0.55990	28.71	92.90
6	Mouse 152 L2 IT ENTm down	0.03931	0.55990	28.71	92.90
7	Mouse 165 L2/3 IT ENTl down	0.03931	0.55990	28.71	92.90
8	Human Inh L2-5 VIP BSPRY down	0.04317	0.55990	6.33	19.88
9	Mouse 148 L2 IT PAR down	0.04412	0.55990	25.12	78.38
10	Mouse 293 CA3 down	0.05366	0.55990	20.09	58.77

Table S2: left cerebral cortex down1(brain up)

Index	Name	P-value	Adjusted p-value	Odds Ratio	Combined score
1	Mouse 33 Sncg down	0.00004230	0.01320	23.65	238.16
2	Human Astro L1-6 FGFR3 AQP1 down	0.0002117	0.02536	3.77	31.88
3	Mouse 359 OPC down	0.0002833	0.02536	3.44	28.12
4	Mouse 19 Pax6 down	0.0003251	0.02536	25.61	205.71
5	Human OPC L1-6 PDGFRA COL20A1 down	0.0004251	0.02652	3.01	23.37
6	Mouse 100 Sst down	0.0005934	0.03086	20.48	152.19
7	Human Inh L1-2 VIP SCML4 down	0.0008709	0.03882	58.00	408.65
8	Human Astro L1-6 FGFR3 PLCG1 down	0.001462	0.05094	2.99	19.52
9	Mouse 318 CT SUB down	0.001598	0.05094	8.60	55.35
10	Mouse 22 Ndnf HPF down	0.001862	0.05094	36.90	231.95

Table S3: right cerebral cortex up1

Index	Name	P-value	Adjusted p-value	Odds Ratio	Combined score
1	Mouse 96 Sst up	0.0001824	0.03374	2.51	21.65
2	Mouse 312 NP SUB up	0.01706	0.64240	10.67	43.42
3	Human Inh L5-6 PVALB ZFPM2-AS1 up	0.02236	0.64240	5.23	19.88
4	Mouse 309 L5 NP CTX up	0.02963	0.64240	40.19	141.43
5	Mouse 318 CT SUB up	0.02963	0.64240	40.19	141.43
6	Mouse 97 Sst up	0.03426	0.64240	1.97	6.66
7	Mouse 314 NP SUB up	0.03931	0.64240	28.71	92.90
8	Human Inh L3-5 SST CDH3 down	0.05686	0.64240	5.39	15.47
9	Mouse 208 L5 IT CTX up	0.05839	0.64240	18.26	51.88
10	Mouse 132 L2/3 IT PPP down	0.05915	0.64240	3.51	9.91

Table S4: right cerebral cortex down1(brain up)

Index	Name	P-value	Adjusted p-value	Odds Ratio	Combined score
1	Mouse 123 DG down	0.000005007	0.001863	23.22	283.42
2	Mouse 293 CA3 down	0.000019440	0.003615	76.90	834.26
3	Mouse 362 Oligo down	0.000038480	0.004772	4.01	40.73
4	Mouse 57 Vip down	0.000119200	0.011090	6.96	62.85
5	Mouse 40 Vip down	0.000231400	0.014930	14.76	123.60
6	Mouse 119 Pvalb Vipr2 down	0.000279500	0.014930	14.01	114.65
7	Mouse 54 Vip down	0.000280900	0.014930	7.28	59.52
8	Mouse 56 Vip down	0.000419900	0.019530	6.73	52.32
9	Human Inh L5-6 PVALB FAM150B down	0.000492400	0.020010	21.95	167.17
10	Mouse 363 Oligo down	0.000538000	0.020010	3.38	25.43
11	Human Inh L6 SST TH down	0.000768200	0.021600	18.62	133.53
12	Mouse 103 Sst up	0.000768200	0.021600	18.62	133.53
13	Mouse 373 SMC down	0.000770800	0.021600	4.00	28.68
14	Mouse 112 Pvalb down	0.000832900	0.021600	18.07	128.13
15	Mouse 71 Sst down	0.000870900	0.021600	58.00	408.65
16	Mouse 33 Sncg down	0.000972600	0.021650	17.07	118.36
17	Mouse 356 Astro down	0.001040000	0.021650	3.53	24.26
18	Mouse 104 Sst down	0.001048000	0.021650	16.60	113.92
19	Human VLMC L1-5 PDGFRA COLEC12 down	0.001199000	0.023480	3.24	21.80
20	Mouse 103 Sst down	0.001478000	0.027490	14.62	95.30
21	Mouse 1 CR down	0.001594000	0.028240	3.12	20.10
22	Mouse 55 Vip down	0.001846000	0.028770	8.25	51.93
23	Mouse 22 Ndnf HPF down	0.001862000	0.028770	36.90	231.95
24	Mouse 300 CA3 down	0.001862000	0.028770	36.90	231.95
25	Human Inh L3-5 VIP IGDCC3 down	0.002122000	0.028770	12.79	78.73

Table S5: hippocampal layer up1

Index	Name	P-value	Adjusted p-value	Odds Ratio	Combined score
1	Mouse 96 Sst up	7.990e-7	0.000142	3.24	45.51
2	Mouse 97 Sst up	0.006042	0.537700	2.39	12.21
3	Mouse 310 L5 NP CTX up	0.009197	0.545700	7.38	34.62
4	Mouse 371 Peri up	0.020430	0.663900	9.65	37.54
5	Mouse 19 Pax6 up	0.021310	0.663900	9.42	36.27
6	Mouse 309 L5 NP CTX up	0.029630	0.663900	40.19	141.43
7	Mouse 318 CT SUB up	0.029630	0.663900	40.19	141.43
8	Mouse 70 Sst up	0.033060	0.663900	7.36	25.11
9	Mouse 314 NP SUB up	0.039310	0.663900	28.71	92.90
10	Human Inh L5-6 SST FBN2 down	0.055560	0.663900	5.47	15.80

Table S6: hippocampal layer down1(brain up)

Index	Name	P-value	Adjusted p-value	Odds Ratio	Combined score
1	Mouse 123 DG down	0.000005007	0.001778	23.22	283.42
2	Mouse 56 Vip down	0.000052470	0.009314	7.98	78.62
3	Mouse 57 Vip down	0.000119200	0.014110	6.96	62.85
4	Mouse 119 Pvalb Vipr2 down	0.000279500	0.023340	14.01	114.65
5	Human VLMC L1-5 PDGFRA COLEC12 down	0.000328800	0.023340	3.58	28.72
6	Human Inh L5-6 PVALB FAM150B down	0.000492400	0.026330	21.95	167.17
7	Mouse 362 Oligo down	0.000582000	0.026330	3.35	24.93
8	Mouse 100 Sst down	0.000593400	0.026330	20.48	152.19
9	Human Inh L6 SST TH down	0.000768200	0.026880	18.62	133.53
10	Mouse 373 SMC down	0.000770800	0.026880	4.00	28.68

Table S7: frontal cortex up1

Index	Name	P-value	Adjusted p-value	Odds Ratio	Combined score
1	Mouse 188 L4/5 IT CTX down	0.001295	0.2450	15.36	102.11
2	Mouse 97 Sst up	0.002250	0.2450	2.61	15.89
3	Mouse 118 Pvalb down	0.004361	0.2450	6.44	34.98
4	Mouse 43 Vip down	0.004974	0.2450	6.19	32.84
5	Mouse 356 Astro up	0.005635	0.2450	8.89	46.03
6	Mouse 44 Vip down	0.006520	0.2450	5.72	28.77
7	Mouse 127 L2/3 IT APr down	0.008008	0.2450	16.22	78.32
8	Mouse 347 L6b CTX up	0.010100	0.2450	7.13	32.75
9	Mouse 102 Sst down	0.010470	0.2450	13.98	63.75
10	Mouse 42 Vip down	0.011050	0.2450	6.88	31.02

Table S8: frontal cortex down1(brain up)

Index	Name	P-value	Adjusted p-value	Odds Ratio	Combined score
1	Mouse 373 SMC down	0.00003329	0.007464	5.02	51.78
2	Mouse 370 Endo down	0.00005046	0.007464	4.42	43.78
3	Mouse 123 DG down	0.00011360	0.007464	17.98	163.35
4	Mouse 57 Vip down	0.00011920	0.007464	6.96	62.85
5	Mouse 363 Oligo down	0.00014290	0.007464	3.71	32.82
6	Mouse 355 V3d down	0.00014340	0.007464	4.23	37.43
7	Mouse 362 Oligo down	0.00015600	0.007464	3.67	32.19
8	Mouse 360 OPC down	0.00023050	0.008104	3.35	28.03
9	Mouse 374 VLMC down	0.00024790	0.008104	4.28	35.57
10	Mouse 356 Astro down	0.00025970	0.008104	3.94	32.49
11	Mouse 376 VLMC down	0.00026610	0.008104	4.67	38.41
12	Human Inh L5-6 PVALB FAM150B down	0.00049240	0.012700	21.95	167.17
13	Mouse 378 Micro down	0.00049280	0.012700	4.76	36.25
14	Mouse 361 Oligo down	0.00061730	0.014770	3.15	23.31
15	Human Inh L6 SST TH down	0.00076820	0.017160	18.62	133.53
16	Mouse 6 Lamp5 Lhx6 down	0.00099360	0.020800	4.82	33.32
17	Human VLMC L1-5 PDGFRA COLEC12 down	0.00119900	0.022420	3.24	21.80
18	Mouse 371 Peri down	0.00120500	0.022420	4.12	27.72
19	Mouse 293 CA3 down	0.00132200	0.023310	45.10	298.98
20	Mouse 358 Astro down	0.00164200	0.027500	3.31	21.23
21	Mouse 366 Oligo down	0.00207000	0.032980	3.01	18.62
22	Mouse 76 Sst down	0.00216600	0.032980	33.82	207.50
23	Mouse 377 Micro down	0.00238500	0.034740	4.11	24.81
24	Mouse 121 DG down	0.00265100	0.037010	4.03	23.90
25	Mouse 56 Vip down	0.00285300	0.038230	5.52	32.33

$$\ell_1 = 3$$

Table S9: left cerebral cortex up2

Index	Name	P-value	Adjusted p-value	Odds Ratio	Combined score
1	Mouse 374 VLMC up	0.02405	0.8553	8.81	32.83
2	Mouse 70 Sst up	0.03306	0.8553	7.36	25.11
3	Human Inh L5-6 PVALB SST CRHR2 down	0.04412	0.8553	25.12	78.38
4	Human Inh L3-5 SST CDH3 down	0.05686	0.8553	5.39	15.47
5	Mouse 293 CA3 up	0.05820	0.8553	2.49	7.08
6	Mouse 15 Lamp5 up	0.05839	0.8553	18.26	51.88
7	Mouse 28 Sncg up	0.06625	0.8553	2.72	7.39
8	Mouse 55 Vip up	0.06779	0.8553	15.45	41.59
9	Mouse 308 L5 NP CTX up	0.06779	0.8553	15.45	41.59
10	Mouse 30 Sncg up	0.07366	0.8553	1.99	5.20

Table S10: left cerebral cortex down2 (brain up)

Index	Name	P-value	Adjusted p-value	Odds Ratio	Combined score
1	Mouse 236 L3 RSP-ACA down	0.0006796	0.14026	67.67	493.57
2	Human Astro L1 FGFR3 SERPINI2 down	0.0011400	0.14022	2.94	19.91
3	Mouse 90 Sst down	0.0032050	0.19350	27.05	155.37
4	Mouse 267 SUB-ProS down	0.0035940	0.19350	25.36	142.75
5	Mouse 91 Sst down	0.0044340	0.19350	22.54	122.14
6	Mouse 379 PVM down	0.0051440	0.19350	3.55	18.72
7	Human Inh L3-6 PAX6 LINC01497 down	0.0068890	0.19350	17.64	87.79
8	Mouse 66 Sst down	0.0085960	0.19350	15.60	74.20
9	Mouse 46 Vip down	0.0089080	0.19350	7.47	35.29
10	Mouse 359 OPC down	0.0093620	0.19350	2.55	11.91

Table S11: right cerebral cortex up2

Index	Name	P-value	Adjusted p-value	Odds Ratio	Combined score
1	Mouse 96 Sst up	0.005729	0.7639	2.00	10.33
2	Mouse 309 L5 NP CTX up	0.029630	0.7639	40.19	141.43
3	Mouse 318 CT SUB up	0.029630	0.7639	40.19	141.43
4	Mouse 314 NP SUB up	0.039310	0.7639	28.71	92.90
5	Mouse 378 Micro up	0.050460	0.7639	5.78	17.27
6	Mouse 208 L5 IT CTX up	0.058390	0.7639	18.26	51.88
7	Mouse 89 Sst up	0.060820	0.7639	5.19	14.52
8	Mouse 313 NP SUB up	0.063100	0.7639	16.74	46.25
9	Mouse 306 L5 NP CTX up	0.063100	0.7639	16.74	46.25
10	Mouse 28 Sncg up	0.066250	0.7639	2.72	7.39

Table S12: right cerebral cortex down2 (brain up)

Index	Name	P-value	Adjusted p-value	Odds Ratio	Combined score
1	Mouse 46 Vip down	0.00006752	0.02093	13.04	125.22
2	Human Inh L5-6 PVALB FAM150B down	0.00049240	0.05025	21.95	167.17
3	Mouse 118 Pvalb down	0.00052920	0.05025	8.19	61.82
4	Mouse 103 Sst up	0.00076820	0.05025	18.62	133.53
5	Mouse 57 Vip down	0.00083500	0.05025	5.87	41.62
6	Mouse 33 Sncg down	0.00097260	0.05025	17.07	118.36
7	Mouse 231 L6 IT CTX down	0.00120900	0.05352	15.75	105.81
8	Mouse 114 Pvalb down	0.00147800	0.05595	14.62	95.30
9	Mouse 306 L5 NP CTX down	0.00167700	0.05595	13.96	89.20
10	Mouse 54 Vip down	0.00206000	0.05595	5.97	36.91

Table S13: hippocampal layer up2

Index	Name	P-value	Adjusted p-value	Odds Ratio	Combined score
1	Mouse 96 Sst up	0.001134	0.1679	2.25	15.27
2	Mouse 30 Sncg up	0.010960	0.7873	2.63	11.86
3	Mouse 371 Peri up	0.020430	0.7873	9.65	37.54
4	Human Exc L6 FEZF2 PDYN up	0.021830	0.7873	2.13	8.14
5	Mouse 97 Sst up	0.034260	0.7873	1.97	6.66
6	Mouse 374 VLMC down	0.043830	0.7873	2.44	7.64
7	Mouse 229 L6 IT CTX up	0.052560	0.7873	2.95	8.70
8	Mouse 231 L6 IT CTX up	0.052990	0.7873	5.62	16.51
9	Human Exc L3 RORB OTOGL up	0.077100	0.7873	13.39	34.32
10	Mouse 31 Sncg up	0.083340	0.7873	2.05	5.10

Table S14: hippocampal layer down2(brain up)

Index	Name	P-value	Adjusted p-value	Odds Ratio	Combined score
1	Mouse 306 L5 NP CTX down	0.00008897	0.01657	19.24	179.47
2	Mouse 57 Vip down	0.00011920	0.01657	6.96	62.85
3	Human Inh L5-6 PVALB FAM150B down	0.00049240	0.03160	21.95	167.17
4	Mouse 118 Pvalb down	0.00052920	0.03160	8.19	61.82
5	Mouse 122 DG down	0.00056830	0.03160	4.65	34.78
6	Mouse 309 L5 NP CTX down	0.00212200	0.08601	12.79	78.73
7	Mouse 76 Sst down	0.00216600	0.08601	33.82	207.50
8	Mouse 121 DG down	0.00265100	0.08813	4.03	23.90
9	Mouse 56 Vip down	0.00285300	0.08813	5.52	32.33
10	Human Inh L1-2 SST CLIC6 down	0.00584800	0.14800	19.32	99.33

Table S15: frontal cortex up2

Index	Name	P-value	Adjusted p-value	Odds Ratio	Combined score
1	Mouse 259 L6 Car3 up	0.0001511	0.02659	34.16	300.54
2	Mouse 158 L2/3 IT ENTl up	0.0040040	0.24800	23.87	131.77
3	Mouse 356 Astro up	0.0056350	0.24800	8.89	46.03
4	Mouse 378 Micro up	0.0056350	0.24800	8.89	46.03
5	Mouse 310 L5 NP CTX up	0.0091970	0.32370	7.38	34.62
6	Mouse 377 Micro up	0.0130900	0.38410	6.45	27.95
7	Mouse 261 L6 Car3 up	0.0162700	0.40900	3.11	12.82
8	Mouse 258 L6 Car3 up	0.0299300	0.62320	7.79	27.33
9	Mouse 373 SMC down	0.0360700	0.62320	2.57	8.53
10	Mouse 141 L3 IT ENTm up	0.0393100	0.62320	28.71	92.90
11	Human Exc L3-5 RORB TNNT2 up	0.0467600	0.62320	6.04	18.50
12	Mouse 260 L6 Car3 up	0.0591500	0.62320	3.51	9.91
13	Mouse 376 VLMC down	0.0606300	0.62320	2.46	6.89
14	Mouse 379 PVM up	0.0648800	0.62320	4.99	13.66
15	Mouse 308 L5 NP CTX up	0.0677900	0.62320	15.45	41.59
16	Mouse 97 Sst up	0.0718300	0.62320	1.77	4.67
17	Mouse 142 L3 IT ENTm up	0.0724600	0.62320	14.35	37.66
18	Mouse 347 L6b CTX up	0.0732500	0.62320	4.65	12.15
19	Human Exc L3 RORB OTOGL up	0.0771000	0.62320	13.39	34.32
20	Mouse 262 L6 Car3 up	0.0798300	0.62320	3.08	7.78
21	Mouse 15 Lamp5 down	0.0817100	0.62320	12.55	31.44
22	Mouse 37 Sncg down	0.0863100	0.62320	11.81	28.94
23	Mouse 358 Astro up	0.0894400	0.62320	4.12	9.96
24	Mouse 185 L2 IT RSP-ACA down	0.0971100	0.62320	2.82	6.57
25	Mouse 329 L6 CT CTX up	0.1045000	0.62320	9.56	21.60

Table S16: frontal cortex down2(brain up)

Index	Name	P-value	Adjusted p-value	Odds Ratio	Combined score
1	Human Inh L5-6 PVALB FAM150B down	0.0004924	0.08099	21.95	167.17
2	Mouse 103 Sst up	0.0007682	0.08099	18.62	133.53
3	Mouse 57 Vip down	0.0008350	0.08099	5.87	41.62
4	Mouse 376 VLMC down	0.0012610	0.08933	4.09	27.32
5	Mouse 113 Pvalb up	0.0017300	0.08933	6.22	39.55
6	Mouse 76 Sst down	0.0021660	0.08933	33.82	207.50
7	Mouse 378 Micro down	0.0023850	0.08933	4.11	24.81
8	Mouse 73 Sst up	0.0028370	0.08933	28.99	170.01
9	Mouse 70 Sst up	0.0029180	0.08933	11.37	66.34
10	Mouse 112 Pvalb up	0.0031970	0.08933	7.05	40.48

$\ell_1 = 2$ and $\ell_1 = 3$

Table S17: left cerebral cortex up

Index	Name	P-value	Adjusted p-value	Odds Ratio	Combined score
1	Mouse 96 Sst up	7.990e-7	0.000172	3.24	45.51
2	Mouse 129 L2/3 IT APr down	0.002728	0.293300	5.58	32.94
3	Human Inh L5-6 PVALB FAM150B down	0.010470	0.469600	13.98	63.75
4	Mouse 132 L2/3 IT PPP down	0.012040	0.469600	4.75	21.00
5	Mouse 125 DG down	0.019890	0.469600	2.97	11.63
6	Human Exc L5 THEMIS LINC01116 up	0.019900	0.469600	1.80	7.04
7	Mouse 120 DG down	0.023570	0.469600	2.85	10.68
8	Mouse 57 Vip down	0.024620	0.469600	3.80	14.06
9	Mouse 53 Vip down	0.026410	0.469600	4.89	17.78
10	Mouse 51 Vip down	0.028580	0.469600	4.74	16.85

Table S18: left cerebral cortex down(brain up)

Index	Name	P-value	Adjusted p-value	Odds Ratio	Combined score
1	Mouse 379 PVM down	0.0002570	0.03358	4.69	38.77
2	Mouse 359 OPC down	0.0002833	0.03358	3.44	28.12
3	Mouse 361 Oligo down	0.0006173	0.03734	3.15	23.31
4	Mouse 360 OPC down	0.0007853	0.03734	3.07	21.94
5	Mouse 356 Astro down	0.0010400	0.03734	3.53	24.26
6	Mouse 374 VLMC down	0.0010790	0.03734	3.81	26.00
7	Mouse 96 Sst up	0.0011340	0.03734	2.25	15.27
8	Mouse 376 VLMC down	0.0012610	0.03734	4.09	27.32
9	Mouse 363 Oligo down	0.0018560	0.04675	3.06	19.23
10	Human Oligo L2-6 OPALIN FTH1P3 down	0.0019730	0.04675	2.88	17.93

Table S19: right cerebral cortex up

Index	Name	P-value	Adjusted p-value	Odds Ratio	Combined score
1	Mouse 96 Sst up	7.990e-7	0.000169	3.24	45.51
2	Human Exc L5 THEMIS LINC01116 up	0.009972	0.539400	1.92	8.84
3	Mouse 6 Lamp5 Lhx6 down	0.020500	0.539400	3.35	13.02
4	Human Exc L5 THEMIS RGPD6 up	0.021510	0.539400	3.30	12.69
5	Human Exc L6 FEZF2 PDYN up	0.021830	0.539400	2.13	8.14
6	Mouse 112 Pvalb up	0.022840	0.539400	5.18	19.59
7	Mouse 309 L5 NP CTX up	0.029630	0.539400	40.19	141.43
8	Mouse 318 CT SUB up	0.029630	0.539400	40.19	141.43
9	Mouse 124 DG down	0.030360	0.539400	3.55	12.40
10	Mouse 97 Sst up	0.034260	0.539400	1.97	6.66

Table S20: right cerebral cortex down(brain up)

Index	Name	P-value	Adjusted p-value	Odds Ratio	Combined score
1	Mouse 267 SUB-ProS down	0.00009371	0.01885	41.00	380.29
2	Mouse 123 DG down	0.00011360	0.01885	17.98	163.35
3	Human Inh L5-6 PVALB FAM150B down	0.00049240	0.05450	21.95	167.17
4	Human Inh L3-6 VIP ZIM2-AS1 down	0.00083290	0.06537	18.07	128.13
5	Mouse 376 VLMC down	0.00126100	0.06537	4.09	27.32
6	Mouse 364 Oligo down	0.00153400	0.06537	3.34	21.67
7	Mouse 22 Ndnf HPF down	0.00186200	0.06537	36.90	231.95
8	Mouse 292 CA3 down	0.00186200	0.06537	36.90	231.95
9	Mouse 294 CA3 down	0.00186200	0.06537	36.90	231.95
10	Mouse 76 Sst down	0.00216600	0.06537	33.82	207.50

Table S21: hippocampal layer up

Index	Name	P-value	Adjusted p-value	Odds Ratio	Combined score
1	Mouse 96 Sst up	0.000002603	0.0005441	3.09	39.70
2	Mouse 97 Sst up	0.000074880	0.0078250	3.29	31.23
3	Human Exc L5 THEMIS LINC01116 up	0.004726000	0.3293000	2.04	10.92
4	Human Exc L5-6 FEZF2 LPO up	0.006643000	0.3471000	2.69	13.49
5	Mouse 358 Astro up	0.013820000	0.4508000	6.31	27.03
6	Mouse 371 Peri up	0.020430000	0.4508000	9.65	37.54
7	Human Exc L5 THEMIS RGPD6 up	0.021510000	0.4508000	3.30	12.69
8	Mouse 309 L5 NP CTX up	0.029630000	0.4508000	40.19	141.43
9	Mouse 318 CT SUB up	0.029630000	0.4508000	40.19	141.43
10	Human Exc L5-6 FEZF2 OR1L8 up	0.034480000	0.4508000	2.13	7.17

Table S22: hippocampal layer down(brain up)

Index	Name	P-value	Adjusted p-value	Odds Ratio	Combined score
1	Mouse 376 VLMC down	0.00005016	0.008295	5.26	52.03
2	Mouse 374 VLMC down	0.00005136	0.008295	4.78	47.17
3	Mouse 378 Micro down	0.00008985	0.009171	5.43	50.59
4	Mouse 123 DG down	0.00011360	0.009171	17.98	163.35
5	Human VLMC L1-5 PDGFRA COLEC12 down	0.00032880	0.017690	3.58	28.72
6	Human Inh L2-5 VIP BSPRY down	0.00033430	0.017690	13.33	106.70
7	Human Inh L3-6 VIP UG0898H09 down	0.00044640	0.017690	22.76	175.61
8	Human Inh L5-6 PVALB FAM150B down	0.00049240	0.017690	21.95	167.17
9	Mouse 377 Micro down	0.00049280	0.017690	4.76	36.25
10	Human Oligo L2-6 OPALIN FTH1P3 down	0.00061100	0.019740	3.16	23.37
11	Mouse 373 SMC down	0.00077080	0.022420	4.00	28.68
12	Human Inh L3-6 VIP ZIM2-AS1 down	0.00083290	0.022420	18.07	128.13
13	Mouse 370 Endo down	0.00091880	0.022830	3.59	25.12
14	Mouse 371 Peri down	0.00120500	0.026340	4.12	27.72
15	Mouse 379 PVM down	0.00122300	0.026340	4.11	27.58
16	Human Astro L1-6 FGFR3 PLCG1 down	0.00146200	0.026910	2.99	19.52
17	Mouse 364 Oligo down	0.00153400	0.026910	3.34	21.67
18	Mouse 363 Oligo down	0.00185600	0.026910	3.06	19.23
19	Mouse 22 Ndnf HPF down	0.00186200	0.026910	36.90	231.95
20	Mouse 292 CA3 down	0.00186200	0.026910	36.90	231.95
21	Mouse 294 CA3 down	0.00186200	0.026910	36.90	231.95
22	Mouse 361 Oligo down	0.00199100	0.026910	2.88	17.88
23	Mouse 366 Oligo down	0.00207000	0.026910	3.01	18.62
24	Human Inh L3-5 VIP IGDCC3 down	0.00212200	0.026910	12.79	78.73
25	Mouse 76 Sst down	0.00216600	0.026910	33.82	207.50

Table S23: frontal cortex up

Index	Name	P-value	Adjusted p-value	Odds Ratio	Combined score
1	Mouse 96 Sst up	2.343e-7	0.000057	3.40	51.90
2	Human Inh L1 PAX6 MIR101-1 up	0.001384	0.167500	14.98	98.61
3	Mouse 301 CA2-IG-FC up	0.003704	0.242200	10.40	58.23
4	Mouse 158 L2/3 IT ENTl up	0.004004	0.242200	23.87	131.77
5	Mouse 378 Micro up	0.005635	0.246200	8.89	46.03
6	Human Inh L2 PAX6 FREM2 up	0.006103	0.246200	3.88	19.79
7	Mouse 302 CA2-IG-FC up	0.007318	0.253000	5.52	27.16
8	Human OPC L1-6 PDGFRA COL20A1 down	0.009788	0.296100	2.32	10.73
9	Mouse 377 Micro up	0.013090	0.334500	6.45	27.95
10	Mouse 358 Astro up	0.013820	0.334500	6.31	27.03

Table S24: frontal cortex down(brain up)

Index	Name	P-value	Adjusted p-value	Odds Ratio	Combined score
1	Mouse 370 Endo down	0.00005046	0.01655	4.42	43.78
2	Mouse 376 VLMC down	0.00026610	0.03233	4.67	38.41
3	Human Inh L5-6 PVALB FAM150B down	0.00049240	0.03233	21.95	167.17
4	Mouse 377 Micro down	0.00049280	0.03233	4.76	36.25
5	Mouse 378 Micro down	0.00049280	0.03233	4.76	36.25
6	Mouse 356 Astro down	0.00104000	0.04918	3.53	24.26
7	Mouse 374 VLMC down	0.00107900	0.04918	3.81	26.00
8	Human VLMC L1-5 PDGFRA COLEC12 down	0.00119900	0.04918	3.24	21.80
9	Human Exc L3-5 RORB RPRM down	0.00158100	0.05762	40.59	261.80
10	Mouse 123 DG down	0.00200500	0.06458	13.06	81.16
11	Mouse 76 Sst down	0.00216600	0.06458	33.82	207.50
12	Human Inh L3-5 VIP HS3ST3A1 down	0.00237000	0.06479	12.28	74.22
13	Human Inh L1-6 PVALB COL15A1 down	0.00306600	0.07736	11.16	64.58
14	Mouse 373 SMC up	0.00387600	0.09080	10.23	56.79
15	Human Astro L1-6 FGFR3 PLCG1 down	0.00449100	0.09374	2.71	14.63
16	Mouse 366 Oligo up	0.00497400	0.09374	6.19	32.84
17	Mouse 57 Vip down	0.00497400	0.09374	4.82	25.56
18	Mouse 379 PVM down	0.00514400	0.09374	3.55	18.72
19	Mouse 363 Oligo down	0.00583900	0.09585	2.75	14.12
20	Human Oligo L2-6 OPALIN FTH1P3 down	0.00584500	0.09585	2.61	13.40
21	Mouse 238 L5 PT CTX up	0.00713700	0.11150	4.40	21.77
22	Human Endo L2-5 NOSTRIN SRGN down	0.00862500	0.12800	2.74	13.01
23	Mouse 359 OPC down	0.00936200	0.12800	2.55	11.91
24	Mouse 373 SMC down	0.01139000	0.12800	3.03	13.57
25	Mouse 137 L2 IT ENTl up	0.01204000	0.12800	6.66	29.43

$\ell_1 = 2$ only

Table S25: left cerebral cortex up1

Index	Name	P-value	Adjusted p-value	Odds Ratio	Combined score
1	Mouse 125 DG down	0.005481	0.6952	3.51	18.26
2	Mouse 129 L2/3 IT APr down	0.015530	0.6952	4.39	18.30
3	Mouse 312 NP SUB up	0.017060	0.6952	10.67	43.42
4	Human Exc L3-5 RORB RPRM up	0.024200	0.6952	3.20	11.91
5	Mouse 124 DG down	0.030360	0.6952	3.55	12.40
6	Mouse 168 L2/3 IT ENTl down	0.037420	0.6952	6.86	22.55
7	Mouse 152 L2 IT ENTm down	0.039310	0.6952	28.71	92.90
8	Mouse 165 L2/3 IT ENTl down	0.039310	0.6952	28.71	92.90
9	Mouse 333 L6b/CT ENT up	0.043170	0.6952	6.33	19.88
10	Mouse 96 Sst up	0.043180	0.6952	1.65	5.19

Table S26: left cerebral cortex down1(brain up)

Index	Name	P-value	Adjusted p-value	Odds Ratio	Combined score
1	Mouse 22 Ndnf HPF down	0.00003345	0.007107	61.52	633.95
2	Mouse 33 Sncg down	0.00004230	0.007107	23.65	238.16
3	Mouse 121 DG down	0.00010380	0.008897	5.32	48.83
4	Mouse 122 DG down	0.00010590	0.008897	5.31	48.58
5	Mouse 125 DG down	0.00028030	0.018210	4.63	37.89
6	Mouse 19 Pax6 down	0.00032510	0.018210	25.61	205.71
7	Mouse 100 Sst down	0.00059340	0.026600	20.48	152.19
8	Mouse 360 OPC down	0.00078530	0.026600	3.07	21.94
9	Mouse 38 Sncg down	0.00083290	0.026600	18.07	128.13
10	Mouse 57 Vip down	0.00083500	0.026600	5.87	41.62

Table S27: right cerebral cortex up1

Index	Name	P-value	Adjusted p-value	Odds Ratio	Combined score
1	Mouse 312 NP SUB up	0.01706	0.6216	10.67	43.42
2	Mouse 70 Sst up	0.03306	0.6216	7.36	25.11
3	Mouse 152 L2 IT ENTm down	0.03931	0.6216	28.71	92.90
4	Mouse 165 L2/3 IT ENTl down	0.03931	0.6216	28.71	92.90
5	Mouse 333 L6b/CT ENT up	0.04317	0.6216	6.33	19.88
6	Mouse 148 L2 IT PAR down	0.04412	0.6216	25.12	78.38
7	Mouse 20 Ndnf HPF up	0.04419	0.6216	3.97	12.37
8	Mouse 293 CA3 down	0.05366	0.6216	20.09	58.77
9	Mouse 132 L2/3 IT PPP down	0.05915	0.6216	3.51	9.91
10	Mouse 125 DG down	0.06211	0.6216	2.44	6.78

Table S28: right cerebral cortex down1(brain up)

Index	Name	P-value	Adjusted p-value	Odds Ratio	Combined score
1	Mouse 100 Sst down	0.00002154	0.006642	28.55	306.79
2	Mouse 33 Sncg down	0.00004230	0.006642	23.65	238.16
3	Mouse 93 Sst down	0.00011090	0.011610	38.44	350.03
4	Mouse 5 Lamp5 Lhx6 down	0.00020960	0.016450	10.12	85.69
5	Mouse 119 Pvalb Vipr2 down	0.00027950	0.017010	14.01	114.65
6	Mouse 19 Pax6 down	0.00032510	0.017010	25.61	205.71
7	Mouse 56 Vip down	0.00041990	0.018840	6.73	52.32
8	Mouse 101 Sst down	0.00063210	0.024810	11.16	82.23
9	Mouse 36 Sncg down	0.00076820	0.026800	18.62	133.53
10	Mouse 71 Sst down	0.00087090	0.027350	58.00	408.65

Table S29: hippocampal layer up1

Index	Name	P-value	Adjusted p-value	Odds Ratio	Combined score
1	Mouse 96 Sst up	0.01187	0.5843	1.88	8.34
2	Mouse 132 L2/3 IT PPP down	0.01204	0.5843	4.75	21.00
3	Mouse 129 L2/3 IT APr down	0.01553	0.5843	4.39	18.30
4	Mouse 276 CA1-ProS up	0.01871	0.5843	10.13	40.31
5	Mouse 19 Pax6 up	0.02131	0.5843	9.42	36.27
6	Mouse 92 Sst up	0.02499	0.5843	8.62	31.80
7	Mouse 372 SMC down	0.02952	0.5843	2.70	9.50
8	Mouse 70 Sst up	0.03306	0.5843	7.36	25.11
9	Mouse 234 L3 RSP-ACA down	0.03332	0.5843	2.41	8.19
10	Mouse 373 SMC down	0.03607	0.5843	2.57	8.53

Table S30: hippocampal layer down1(brain up)

Index	Name	P-value	Adjusted p-value	Odds Ratio	Combined score
1	Mouse 100 Sst down	0.00002154	0.007488	28.55	306.79
2	Mouse 33 Sncg down	0.00004230	0.007488	23.65	238.16
3	Mouse 93 Sst down	0.00011090	0.013080	38.44	350.03
4	Mouse 366 Oligo down	0.00016390	0.014510	3.65	31.84
5	Mouse 5 Lamp5 Lhx6 down	0.00020960	0.014840	10.12	85.69
6	Mouse 119 Pvalb Vipr2 down	0.00027950	0.016440	14.01	114.65
7	Mouse 19 Pax6 down	0.00032510	0.016440	25.61	205.71
8	Mouse 56 Vip down	0.00041990	0.018580	6.73	52.32
9	Mouse 362 Oligo down	0.00058200	0.022020	3.35	24.93
10	Mouse 101 Sst down	0.00063210	0.022020	11.16	82.23

Table S31: frontal cortex up1

Index	Name	P-value	Adjusted p-value	Odds Ratio	Combined score
1	Mouse 118 Pvalb down	0.0005292	0.05362	8.19	61.82
2	Mouse 75 Sst down	0.0005414	0.05362	21.19	159.39
3	Mouse 86 Sst down	0.0010850	0.05362	50.74	346.39
4	Mouse 106 Pvalb down	0.0012090	0.05362	15.75	105.81
5	Mouse 117 Pvalb down	0.0012090	0.05362	15.75	105.81
6	Mouse 326 L6 CT CTX down	0.0013220	0.05362	45.10	298.98
7	Mouse 114 Pvalb down	0.0014780	0.05362	14.62	95.30
8	Mouse 129 L2/3 IT APr down	0.0027280	0.08141	5.58	32.94
9	Mouse 95 Sst down	0.0032050	0.08141	27.05	155.37
10	Mouse 116 Pvalb down	0.0032050	0.08141	27.05	155.37

Table S32: frontal cortex down1(brain up)

Index	Name	P-value	Adjusted p-value	Odds Ratio	Combined score
1	Mouse 288 CA1 down	0.0008709	0.09914	58.00	408.65
2	Mouse 276 CA1-ProS down	0.0013220	0.09914	45.10	298.98
3	Mouse 293 CA3 down	0.0013220	0.09914	45.10	298.98
4	Mouse 88 Sst up	0.0023700	0.12890	12.28	74.22
5	Mouse 90 Sst up	0.0035380	0.12890	10.58	59.72
6	Mouse 95 Sst up	0.0040040	0.12890	23.87	131.77
7	Mouse 87 Sst up	0.0044340	0.12890	22.54	122.14
8	Mouse 297 CA3 down	0.0053560	0.12890	20.29	106.08
9	Mouse 361 Oligo down	0.0058920	0.12890	2.60	13.37
10	Mouse 362 Oligo down	0.0062040	0.12890	2.72	13.82

$\ell_1 = 3$ only

Table S33: left cerebral cortex up2

Index	Name	P-value	Adjusted p-value	Odds Ratio	Combined score
1	Mouse 70 Sst up	0.002918	0.5107	11.37	66.34
2	Mouse 261 L6 Car3 up	0.016270	0.8030	3.11	12.82
3	Mouse 72 Sst up	0.022840	0.8030	5.18	19.59
4	Mouse 235 L3 RSP-ACA up	0.026920	0.8030	8.27	29.89
5	Mouse 302 CA2-IG-FC up	0.041460	0.8030	4.07	12.96
6	Human Inh L5-6 PVALB SST CRHR2 down	0.044120	0.8030	25.12	78.38
7	Mouse 182 L2/3 IT CTX up	0.054270	0.8030	5.54	16.15
8	Human Inh L3-5 SST CDH3 down	0.056860	0.8030	5.39	15.47
9	Mouse 293 CA3 up	0.058200	0.8030	2.49	7.08
10	Mouse 15 Lamp5 up	0.058390	0.8030	18.26	51.88

Table S34: left cerebral cortex down2(brain up)

Index	Name	P-value	Adjusted p-value	Odds Ratio	Combined score
1	Mouse 8 Lamp5 down	0.0004032	0.06473	23.64	184.78
2	Mouse 314 NP SUB down	0.0004464	0.06473	22.76	175.61
3	Mouse 300 CA3 down	0.0018620	0.13710	36.90	231.95
4	Human Inh L1 VIP KLHDC8B down	0.0018910	0.13710	13.35	83.70
5	Mouse 299 CA3 down	0.0024910	0.14450	31.22	187.17
6	Mouse 119 Pvalb Vipr2 down	0.0038760	0.17710	10.23	56.79
7	Mouse 332 L6 CT ENTm down	0.0048850	0.17710	21.35	113.64
8	Mouse 10 Lamp5 down	0.0048850	0.17710	21.35	113.64
9	Mouse 296 CA3 down	0.0085960	0.25790	15.60	74.20
10	Mouse 46 Vip down	0.0089080	0.25790	7.47	35.29
11	Mouse 121 DG down	0.0109700	0.25790	3.41	15.37
12	Mouse 110 Pvalb down	0.0118100	0.25790	13.08	58.06
13	Human Inh L1 LAMP5 RAB11FIP1 down	0.0125100	0.25790	12.67	55.51
14	Human Inh L6 SST TH down	0.0139600	0.25790	11.92	50.93
15	Mouse 112 Pvalb down	0.0147100	0.25790	11.58	48.87
16	Mouse 56 Vip down	0.0160700	0.25790	4.35	17.95
17	Mouse 33 Sncg down	0.0162600	0.25790	10.96	45.13
18	Human Inh L5-6 LAMP5 CRABP1 down	0.0170600	0.25790	10.67	43.42
19	Mouse 106 Pvalb down	0.0187100	0.25790	10.13	40.31
20	Mouse 3 Lamp5 Lhx6 down	0.0187100	0.25790	10.13	40.31
21	Mouse 353 NP PPP down	0.0195600	0.25790	9.89	38.89
22	Mouse 103 Sst down	0.0213100	0.25790	9.42	36.27
23	Mouse 114 Pvalb down	0.0213100	0.25790	9.42	36.27
24	Human Inh L2-5 VIP SOX11 up	0.0218700	0.25790	5.27	20.16
25	Mouse 302 CA2-IG-FC down	0.0231200	0.25790	9.00	33.92

Table S35: right cerebral cortex up2

Index	Name	P-value	Adjusted p-value	Odds Ratio	Combined score
1	Mouse 375 VLMC down	0.05182	0.8934	2.34	6.92
2	Mouse 261 L6 Car3 up	0.05307	0.8934	2.56	7.51
3	Human Inh L5-6 SST PIK3CD down	0.06082	0.8934	5.19	14.52
4	Mouse 89 Sst up	0.06082	0.8934	5.19	14.52
5	Mouse 28 Sncg up	0.06625	0.8934	2.72	7.39
6	Mouse 330 L6 CT CTX down	0.07710	0.8934	13.39	34.32
7	Mouse 238 L5 PT CTX down	0.08171	0.8934	12.55	31.44
8	Mouse 142 L3 IT ENTm down	0.09088	0.8934	11.16	26.76
9	Mouse 259 L6 Car3 up	0.09996	0.8934	10.04	23.12
10	Mouse 308 L5 NP CTX down	0.10450	0.8934	9.56	21.60

Table S36: right cerebral cortex down2(brain up)

Index	Name	P-value	Adjusted p-value	Odds Ratio	Combined score
1	Mouse 10 Lamp5 down	0.0001511	0.03598	34.16	300.54
2	Mouse 8 Lamp5 down	0.0004032	0.03598	23.64	184.78
3	Mouse 314 NP SUB down	0.0004464	0.03598	22.76	175.61
4	Mouse 118 Pvalb down	0.0005292	0.03598	8.19	61.82
5	Mouse 144 L3 IT ENTl down	0.0010850	0.05479	50.74	346.39
6	Mouse 3 Lamp5 Lhx6 down	0.0012090	0.05479	15.75	105.81
7	Mouse 300 CA3 down	0.0018620	0.07237	36.90	231.95
8	Mouse 140 L3 IT ENTm down	0.0021660	0.07364	33.82	207.50
9	Mouse 299 CA3 down	0.0024910	0.07528	31.22	187.17
10	Mouse 56 Vip down	0.0028530	0.07761	5.52	32.33

Table S37: hippocampal layer up2

Index	Name	P-value	Adjusted p-value	Odds Ratio	Combined score
1	Mouse 259 L6 Car3 up	0.004885	0.7768	21.35	113.64
2	Mouse 9 Lamp5 up	0.023120	0.8555	9.00	33.92
3	Mouse 374 VLMC down	0.043830	0.8555	2.44	7.64
4	Mouse 10 Lamp5 up	0.051720	0.8555	5.70	16.88
5	Mouse 229 L6 IT CTX up	0.052560	0.8555	2.95	8.70
6	Mouse 231 L6 IT CTX up	0.052990	0.8555	5.62	16.51
7	Mouse 261 L6 Car3 up	0.053070	0.8555	2.56	7.51
8	Mouse 28 Sncg up	0.066250	0.8555	2.72	7.39
9	Mouse 55 Vip up	0.067790	0.8555	15.45	41.59
10	Human Exc L3 RORB OTOGL up	0.077100	0.8555	13.39	34.32
11	Mouse 262 L6 Car3 up	0.079830	0.8555	3.08	7.78
12	Human Exc L3-5 RORB RPRM up	0.081620	0.8555	2.53	6.33
13	Mouse 355 V3d up	0.081620	0.8555	2.53	6.33
14	Mouse 370 Endo down	0.083840	0.8555	2.05	5.08
15	Mouse 373 SMC down	0.098590	0.8555	2.11	4.89
16	Mouse 368 Oligo down	0.103200	0.8555	1.93	4.38
17	Mouse 329 L6 CT CTX up	0.104500	0.8555	9.56	21.60
18	Human Exc L5 RORB MED8 up	0.131000	0.8555	7.43	15.11
19	Mouse 8 Lamp5 up	0.139700	0.8555	6.92	13.62
20	Mouse 379 PVM down	0.159100	0.8555	1.95	3.59
21	Mouse 30 Sncg up	0.159500	0.8555	1.69	3.10
22	Mouse 233 L6 IT ENTl up	0.161000	0.8555	5.90	10.78
23	Mouse 118 Pvalb up	0.165200	0.8555	5.73	10.32
24	Mouse 60 Sst Chodl up	0.173600	0.8555	5.42	9.50
25	Human Inh L1-6 LAMP5 CA1 up	0.181800	0.8555	5.14	8.77

Table S38: hippocampal layer down2(brain up)

Index	Name	P-value	Adjusted p-value	Odds Ratio	Combined score
1	Mouse 314 NP SUB down	0.0004464	0.06138	22.76	175.61
2	Mouse 118 Pvalb down	0.0005292	0.06138	8.19	61.82
3	Mouse 306 L5 NP CTX down	0.0016770	0.12970	13.96	89.20
4	Mouse 56 Vip down	0.0028530	0.16490	5.52	32.33
5	Mouse 142 L3 IT ENTm down	0.0040040	0.16490	23.87	131.77
6	Mouse 10 Lamp5 down	0.0048850	0.16490	21.35	113.64
7	Mouse 57 Vip down	0.0049740	0.16490	4.82	25.56
8	Mouse 121 DG down	0.0109700	0.28630	3.41	15.37
9	Mouse 122 DG down	0.0111100	0.28630	3.40	15.29
10	Mouse 120 DG down	0.0235700	0.35620	2.85	10.68

Table S39: frontal cortex up2

Index	Name	P-value	Adjusted p-value	Odds Ratio	Combined score
1	Mouse 259 L6 Car3 up	0.004885	0.6287	21.35	113.64
2	Mouse 31 Sncg down	0.010470	0.6287	13.98	63.75
3	Mouse 20 Ndnf HPF down	0.011130	0.6287	13.52	60.80
4	Mouse 373 SMC down	0.036070	0.6287	2.57	8.53
5	Mouse 141 L3 IT ENTm up	0.039310	0.6287	28.71	92.90
6	Mouse 261 L6 Car3 up	0.053070	0.6287	2.56	7.51
7	Mouse 376 VLMC down	0.060630	0.6287	2.46	6.89
8	Human Oligo L2-6 OPALIN FTH1P3 up	0.062160	0.6287	5.12	14.22
9	Mouse 308 L5 NP CTX up	0.067790	0.6287	15.45	41.59
10	Mouse 310 L5 NP CTX up	0.069020	0.6287	4.81	12.87
11	Mouse 142 L3 IT ENTm up	0.072460	0.6287	14.35	37.66
12	Human Exc L3 RORB OTOGL up	0.077100	0.6287	13.39	34.32
13	Mouse 351 L6b CTX down	0.077100	0.6287	13.39	34.32
14	Mouse 262 L6 Car3 up	0.079830	0.6287	3.08	7.78
15	Mouse 15 Lamp5 down	0.081710	0.6287	12.55	31.44
16	Mouse 37 Sncg down	0.086310	0.6287	11.81	28.94
17	Mouse 158 L2/3 IT ENTl up	0.090880	0.6287	11.16	26.76
18	Mouse 185 L2 IT RSP-ACA down	0.097110	0.6287	2.82	6.57
19	Mouse 329 L6 CT CTX up	0.104500	0.6287	9.56	21.60
20	Mouse 140 L3 IT ENTm up	0.117800	0.6287	8.37	17.89
21	Mouse 258 L6 Car3 down	0.131000	0.6287	7.43	15.11
22	Mouse 30 Sncg down	0.131000	0.6287	7.43	15.11
23	Mouse 8 Lamp5 down	0.135400	0.6287	7.17	14.34
24	Mouse 97 Sst up	0.137600	0.6287	1.58	3.13
25	Human Inh L3-6 VIP UG0898H09 down	0.139700	0.6287	6.92	13.62

Table S40: frontal cortex down2(brain up)

Index	Name	P-value	Adjusted p-value	Odds Ratio	Combined score
1	Mouse 112 Pvalb up	0.0003551	0.06854	8.98	71.30
2	Mouse 110 Pvalb up	0.0043500	0.31430	3.07	16.67
3	Mouse 116 Pvalb up	0.0048850	0.31430	21.35	113.64
4	Mouse 113 Pvalb up	0.0109300	0.38000	4.89	22.10
5	Mouse 111 Pvalb up	0.0110400	0.38000	3.05	13.75
6	Mouse 115 Pvalb up	0.0118100	0.38000	13.08	58.06
7	Mouse 109 Pvalb up	0.0142200	0.39200	4.51	19.20
8	Mouse 355 V3d up	0.0242000	0.45970	3.20	11.91
9	Mouse 57 Vip down	0.0246200	0.45970	3.80	14.06
10	Mouse 105 Pvalb up	0.0259500	0.45970	8.44	30.82
11	Mouse 114 Pvalb up	0.0274900	0.45970	4.82	17.31
12	Mouse 118 Pvalb down	0.0285800	0.45970	4.74	16.85
13	Mouse 70 Sst up	0.0330600	0.49080	7.36	25.11
14	Mouse 80 Sst up	0.0467600	0.57230	6.04	18.50
15	Mouse 10 Lamp5 up	0.0517200	0.57230	5.70	16.88
16	Mouse 334 L6b/CT ENT down	0.0536600	0.57230	20.09	58.77
17	Human Inh L3-5 SST CDH3 down	0.0568600	0.57230	5.39	15.47
18	Mouse 98 Sst up	0.0632200	0.57230	3.41	9.41
19	Human Exc L6 THEMIS SLN up	0.0657300	0.57230	3.35	9.12
20	Human Oligo L5-6 OPALIN LDLRAP1 up	0.0688000	0.57230	2.69	7.19
21	Human Exc L6 THEMIS LINC00343 up	0.0690200	0.57230	4.81	12.87
22	Mouse 97 Sst up	0.0718300	0.57230	1.77	4.67
23	Mouse 142 L3 IT ENTm up	0.0724600	0.57230	14.35	37.66
24	Mouse 56 Vip down	0.0726200	0.57230	3.21	8.41
25	Mouse 73 Sst up	0.0771000	0.57230	13.39	34.32
