# Supplementary material for: Somatostatin-Expressing Neurons Regulate Sleep Deprivation and Recovery
Source: Genes (Basel). 2026 Jan 1;17(1):51. doi: 10.3390/genes17010051 (PMC12840664; doi:10.3390/genes17010051)
Supplement: Supplementary file 1 [file genes-17-00051-s001.zip › Supplementary File S6-Supplemetrary_Figures.pdf]

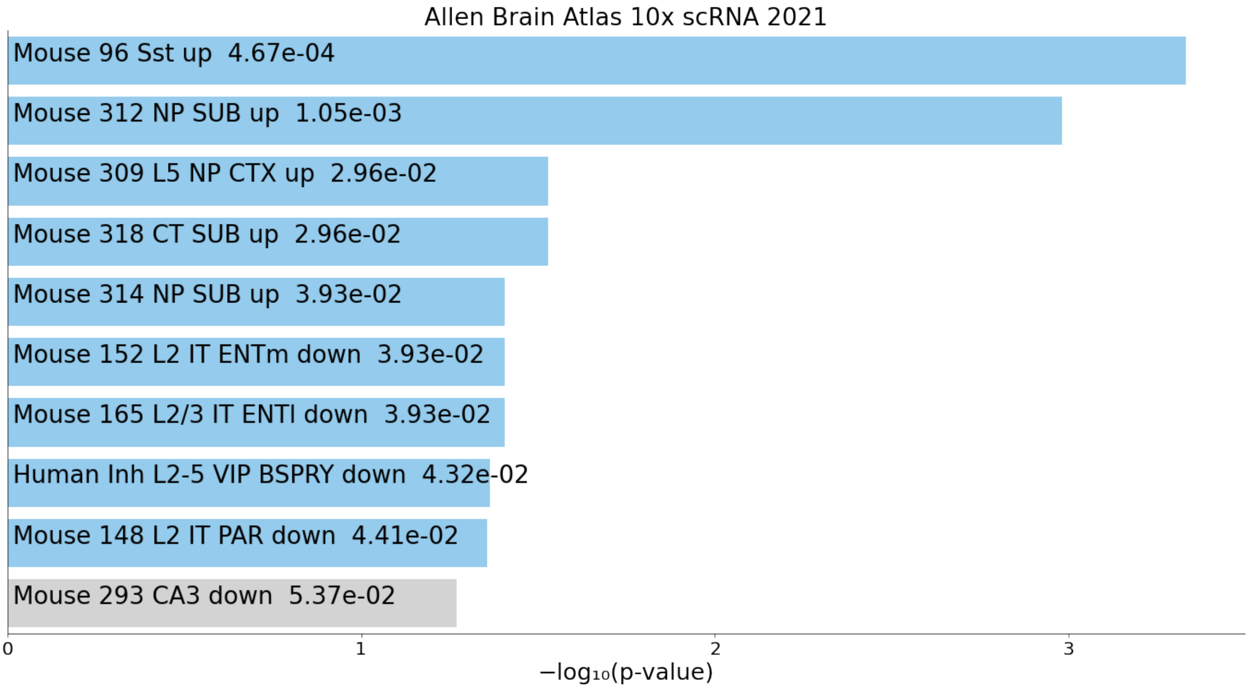

Figure S2 left_cerebral_cortex_up1

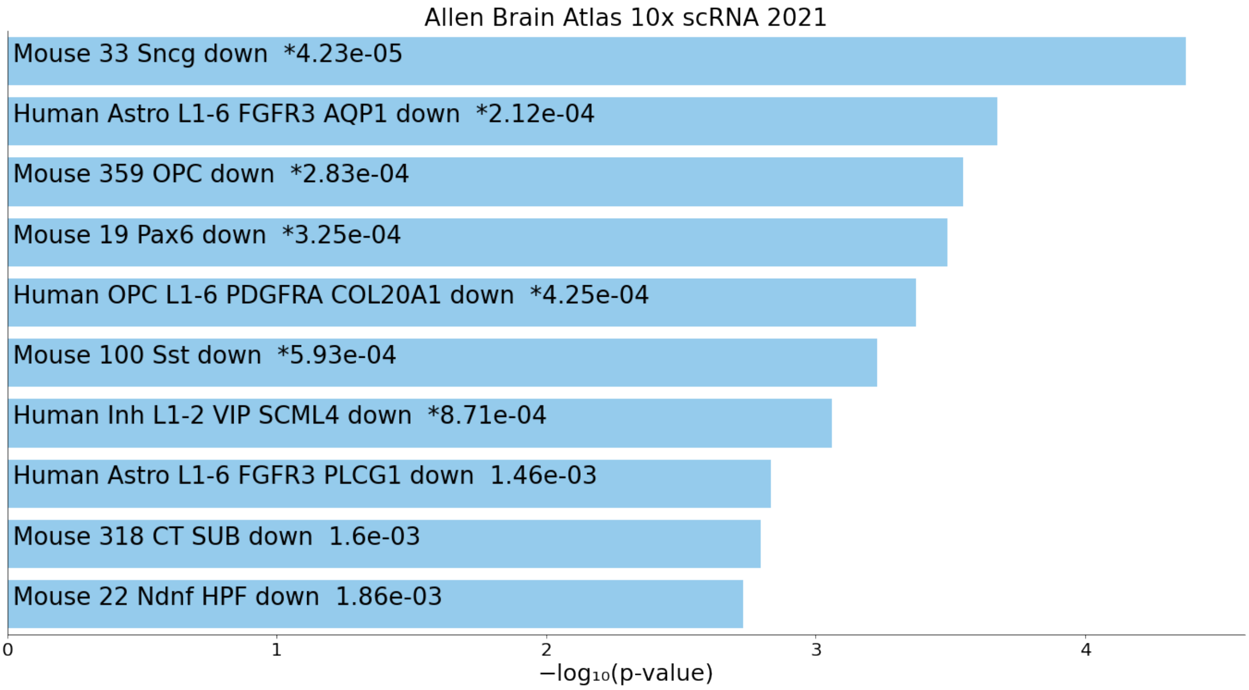

Figure S3 left_cerebral_cortex_down1(brain)

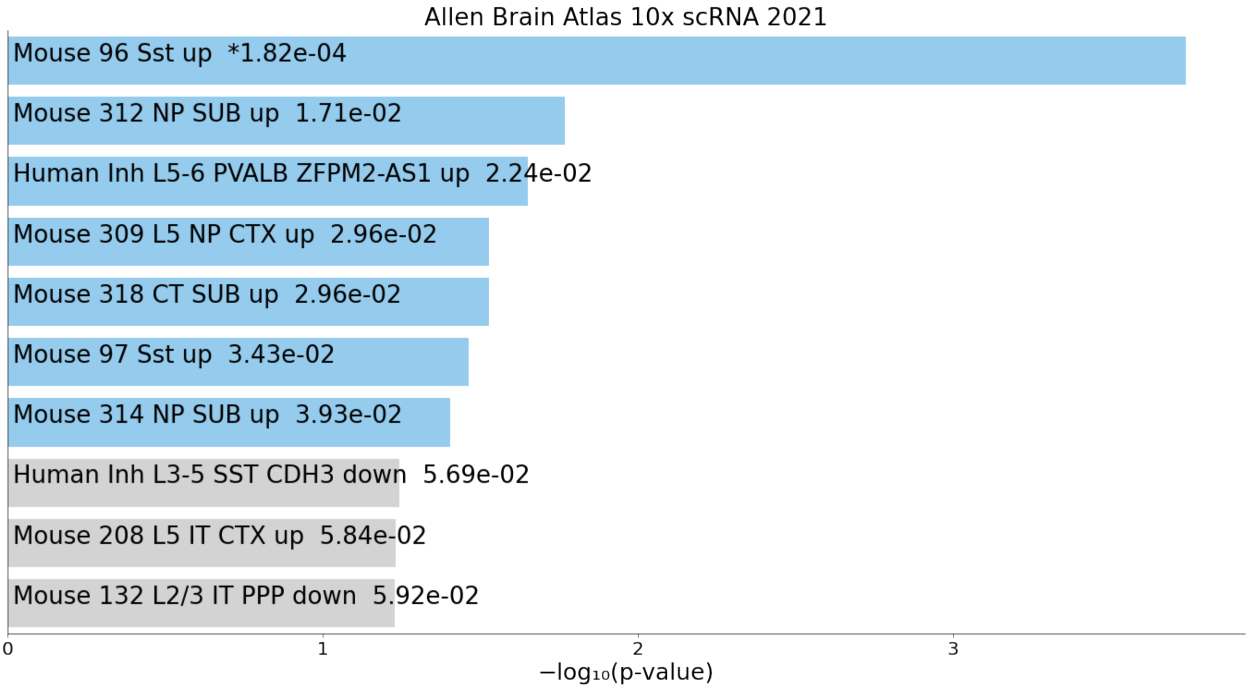

Figure S4 right_cerebral_cortex_up1

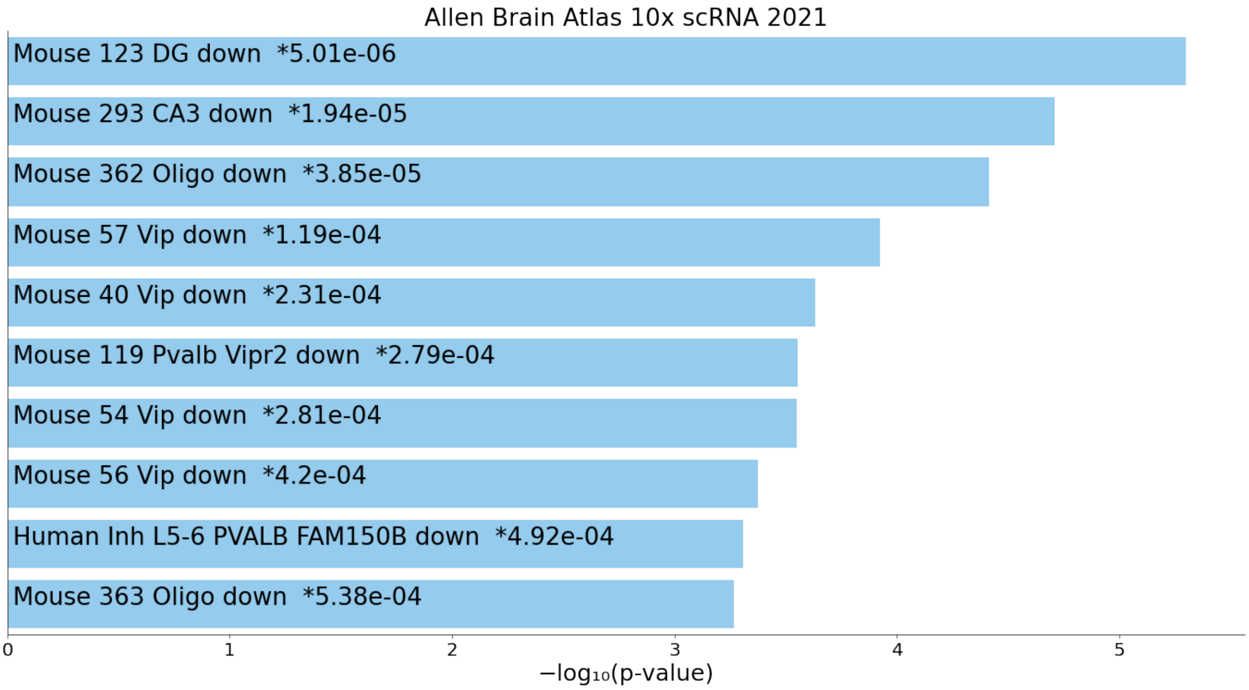

Figure S5 right_cerebral_cortex_down1(brain)

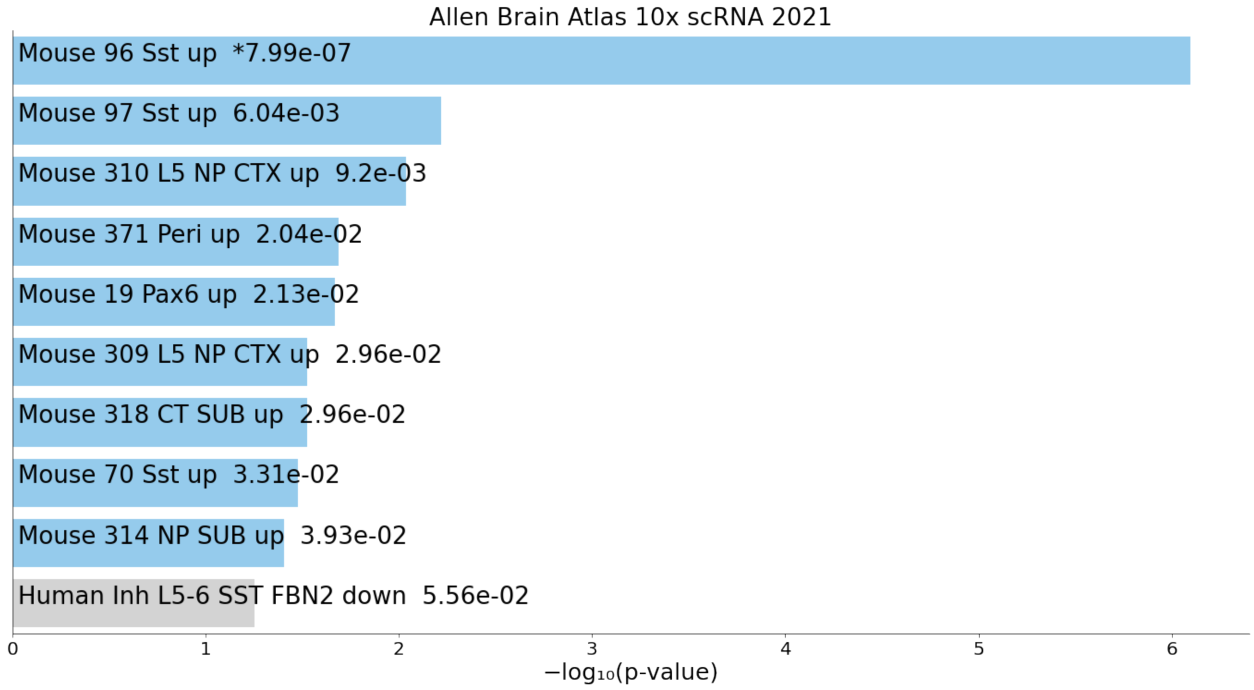

Figure S6 hippocampal_layer_up1

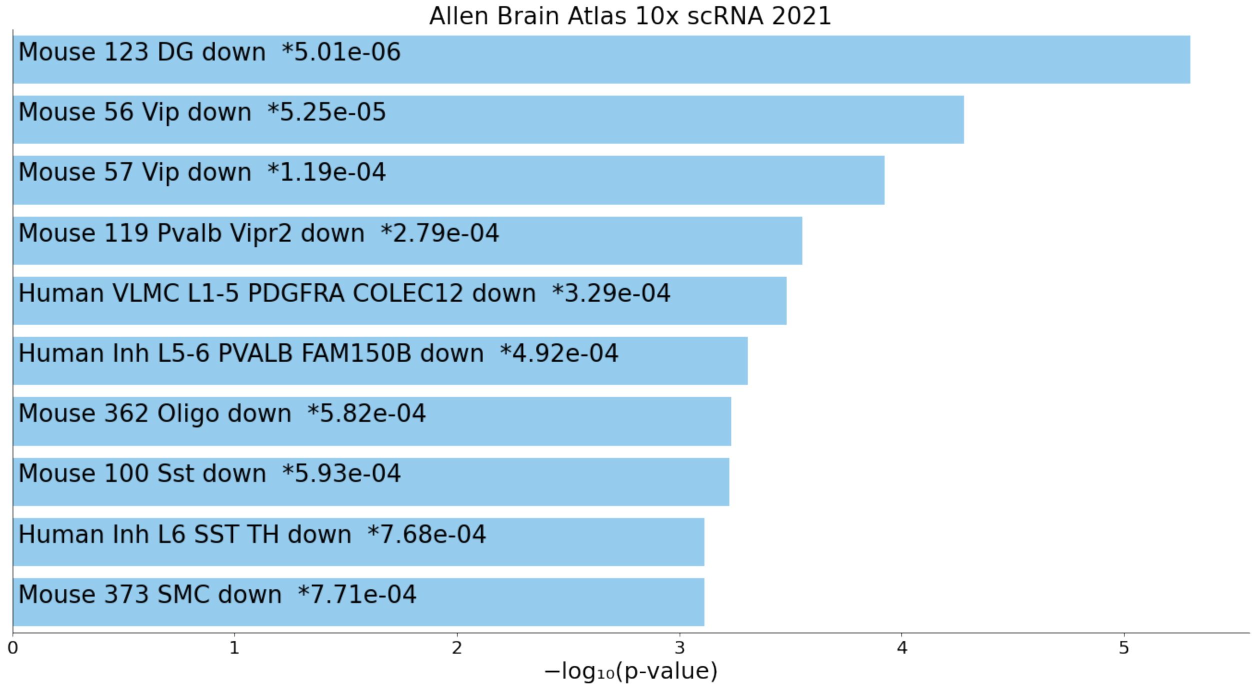

Figure S7 hippocampal_layer_down1(brain up)

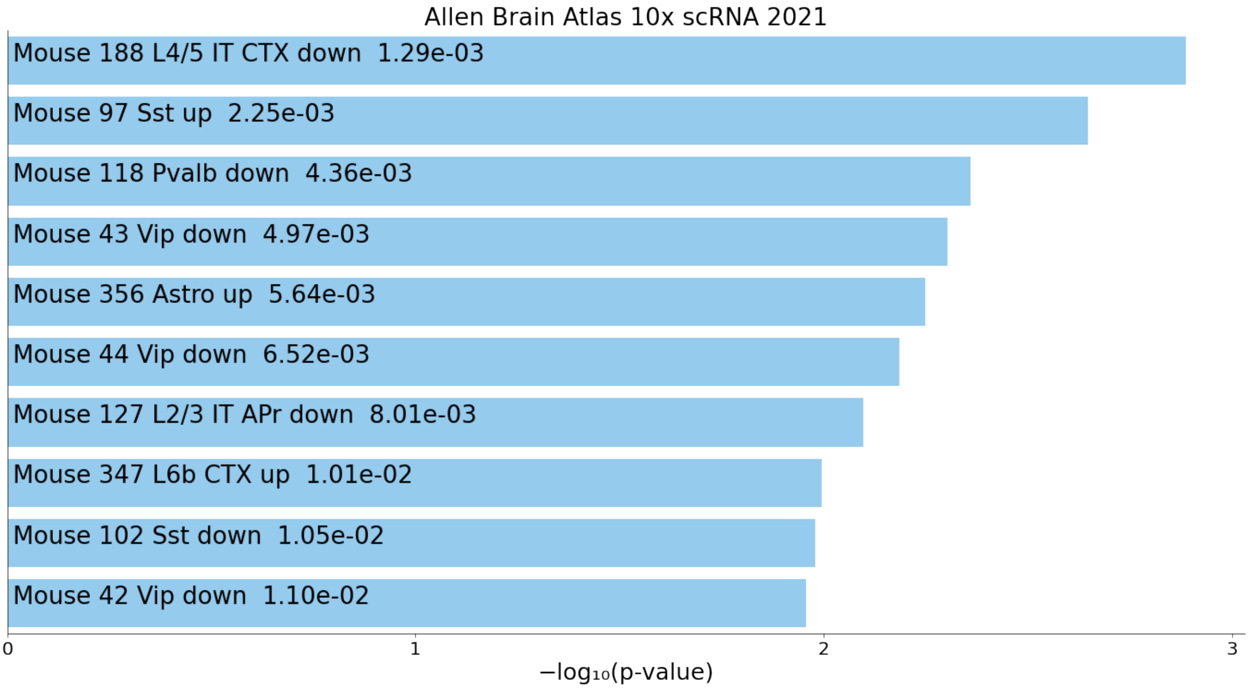

Figure S8 frontal_cortex_up1

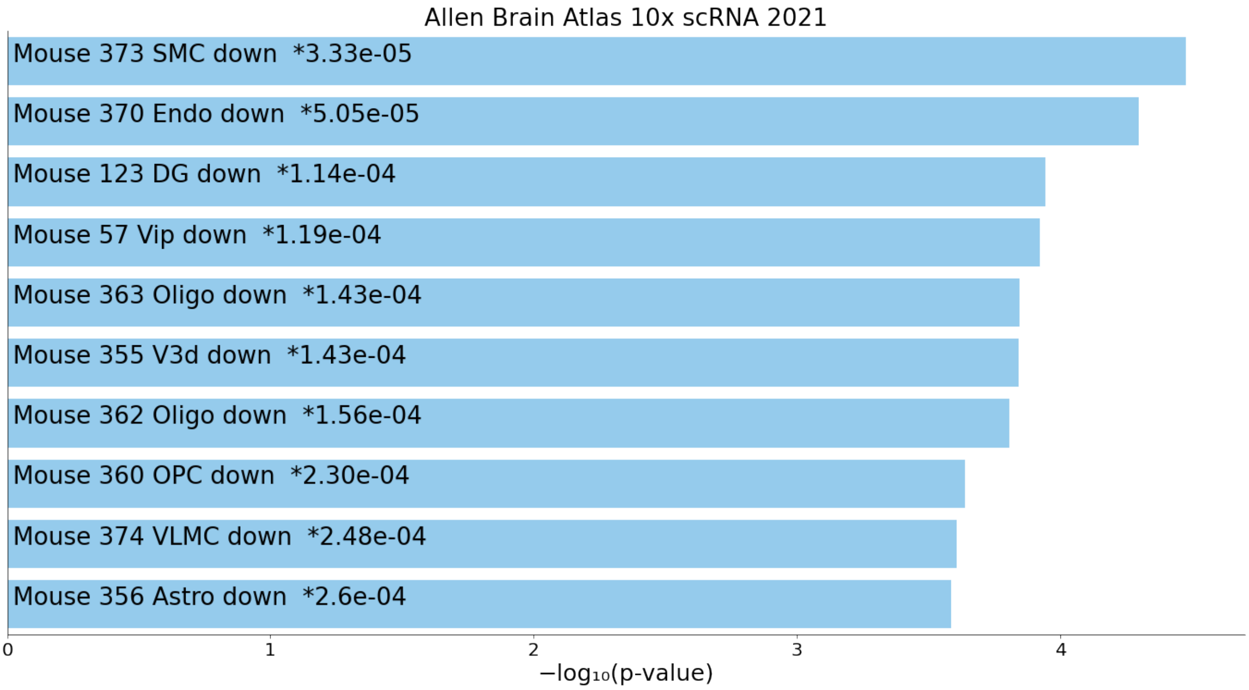

Figure S9 frontal_cortex_down1(brain up)

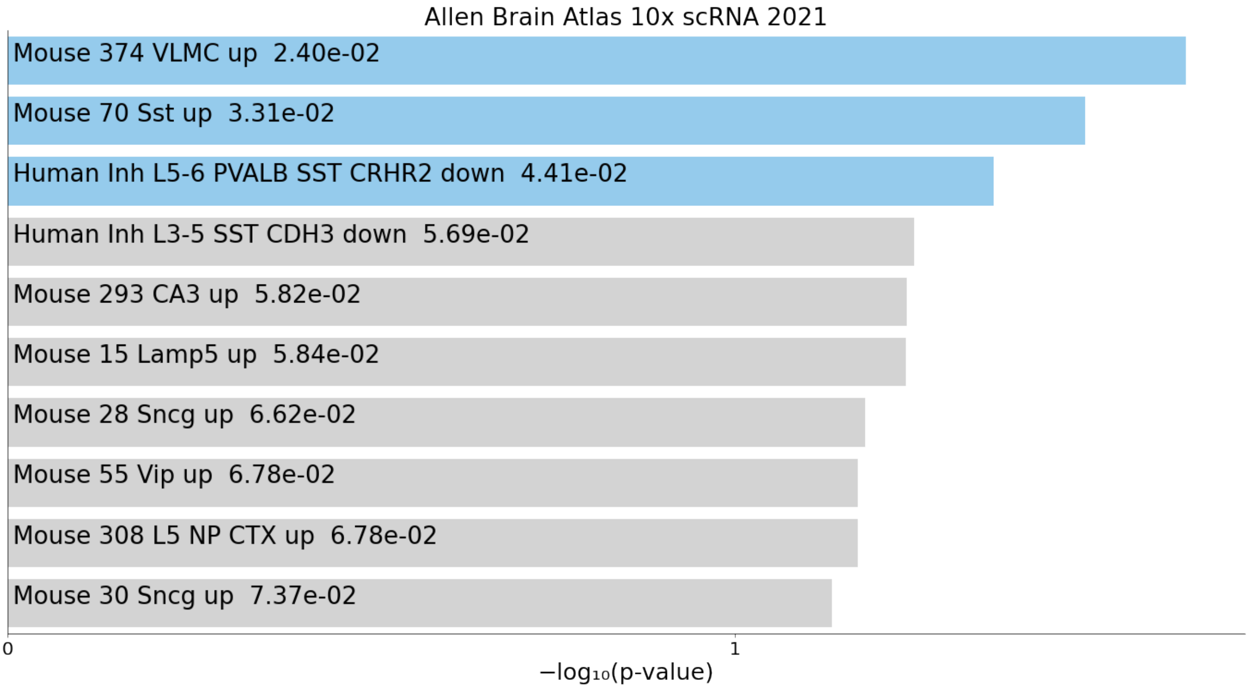

Figure S10 left_cerebral_cortex_up2

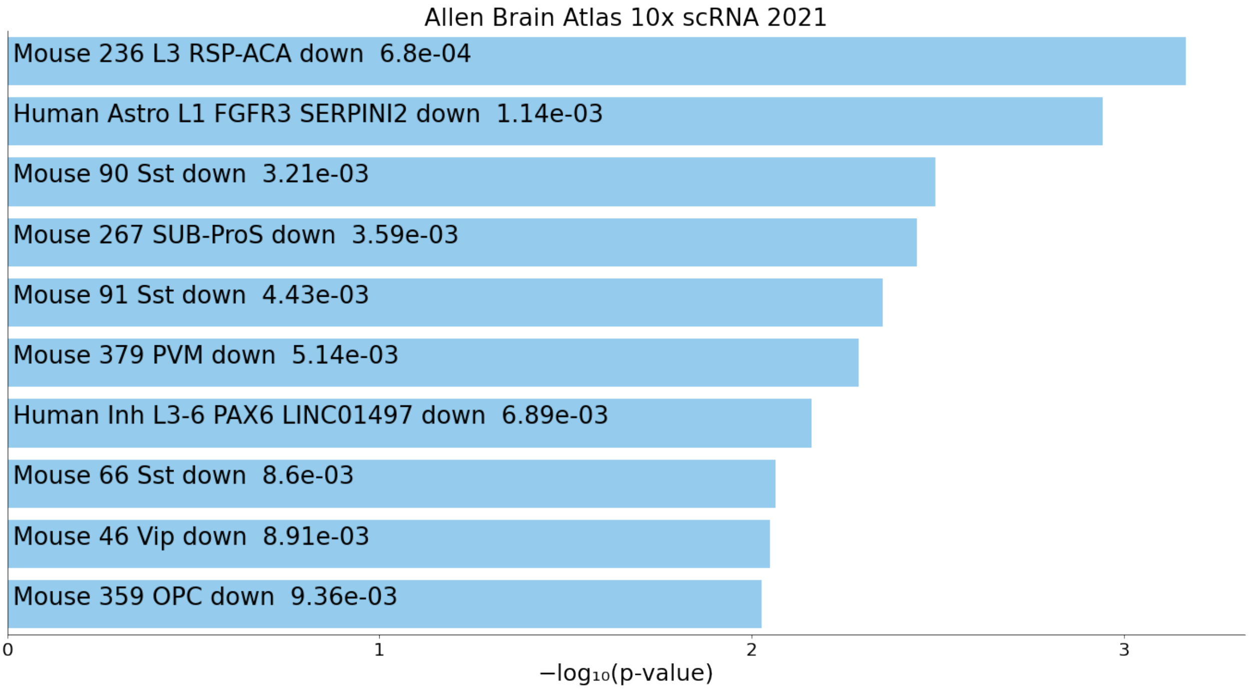

Figure S11 left_cerebral_cortex_down2(brain up)

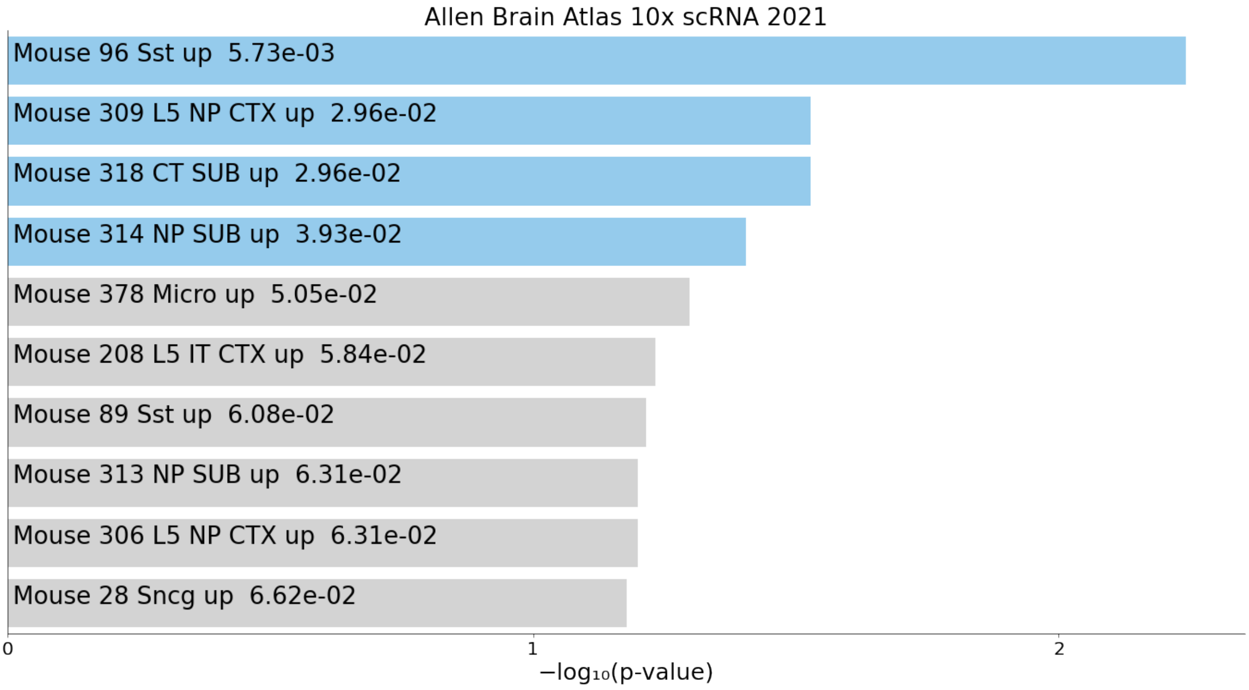

Figure S12 right_cerebral_cortex_up2

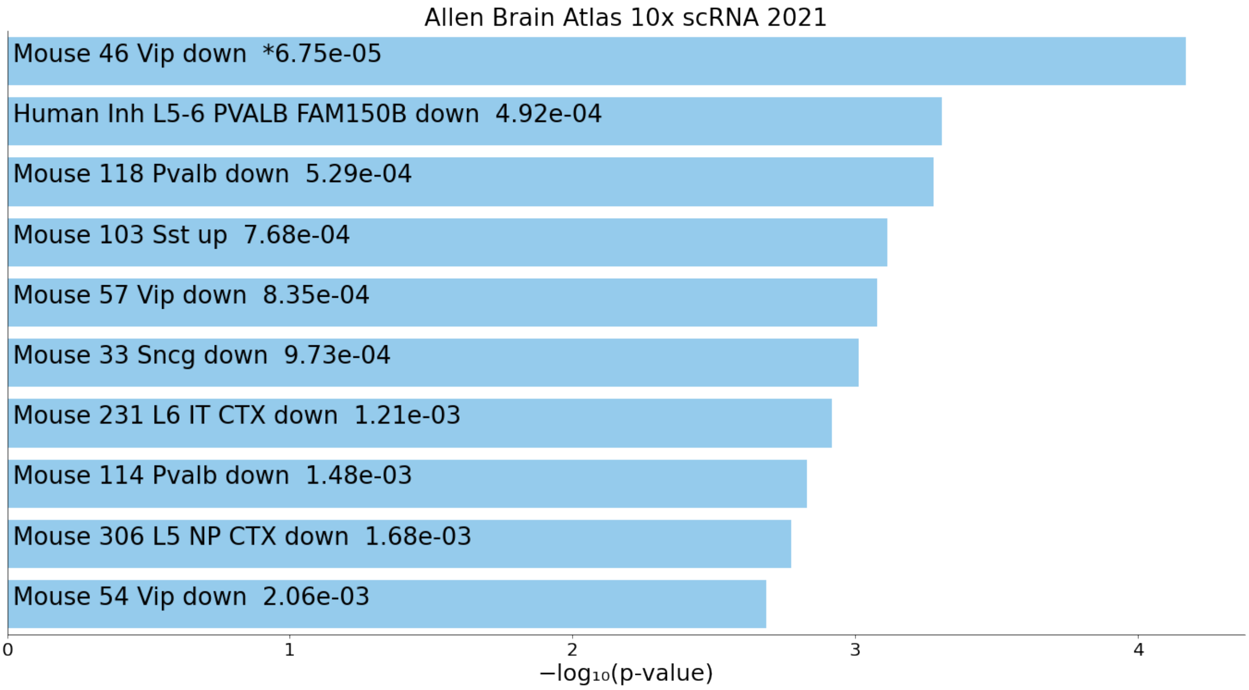

Figure S13 right_cerebral_cortex_down2(brain up)

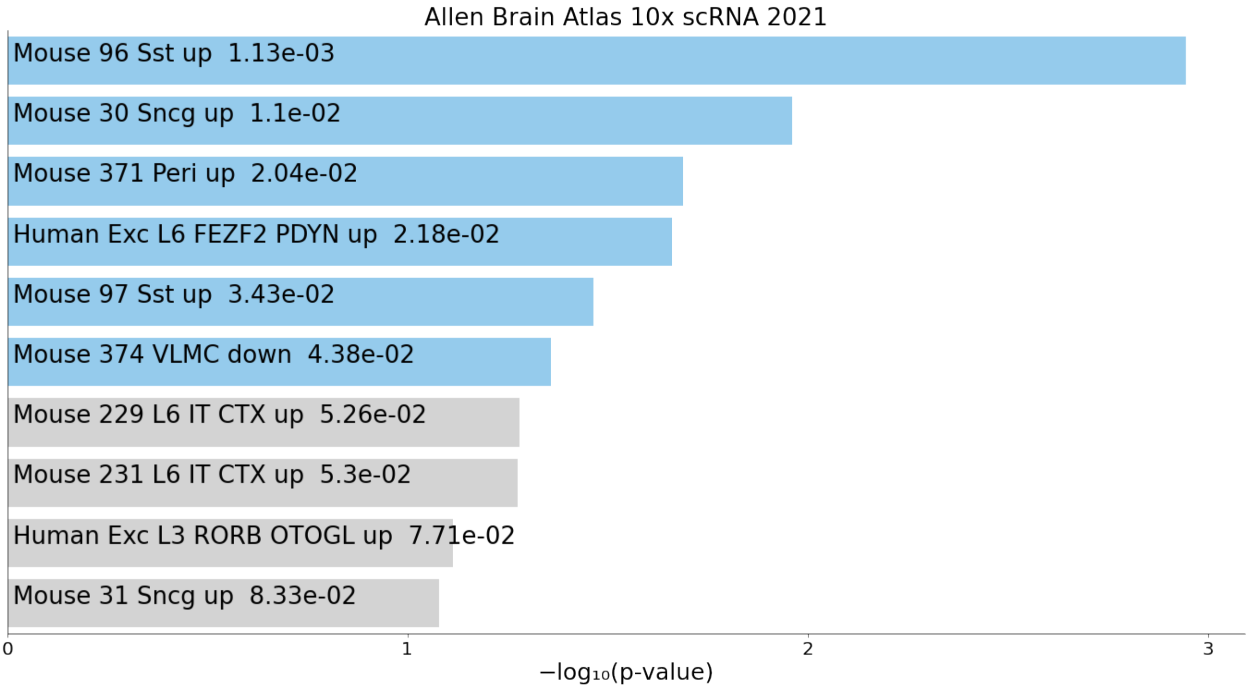

Figure S14 hippocampal_layer_up2

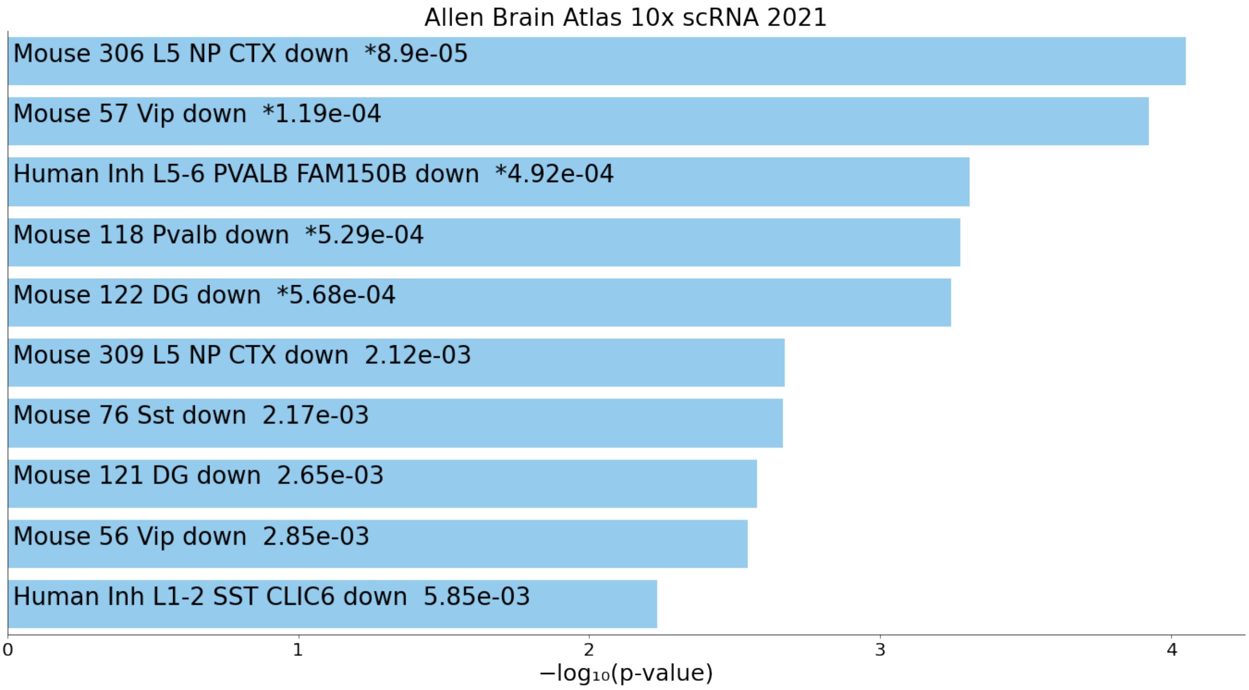

Figure S15 hippocampal_layer_down2(brain up)

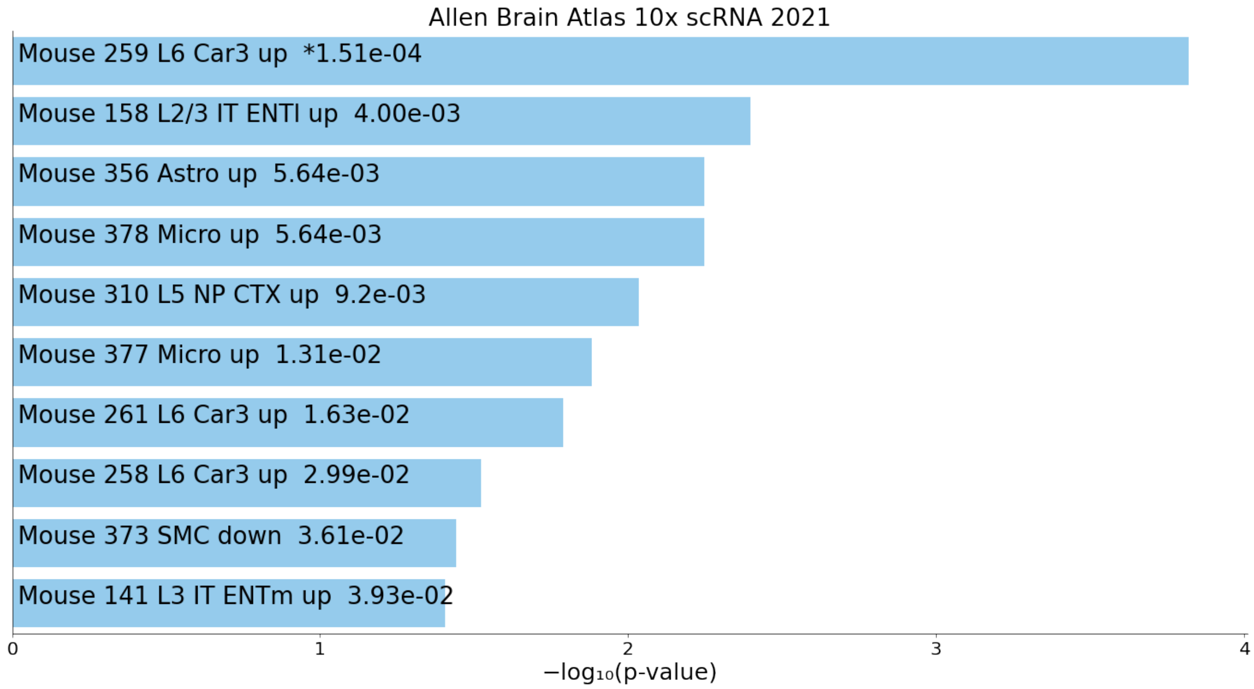

Figure S16 frontal_cortex_up2

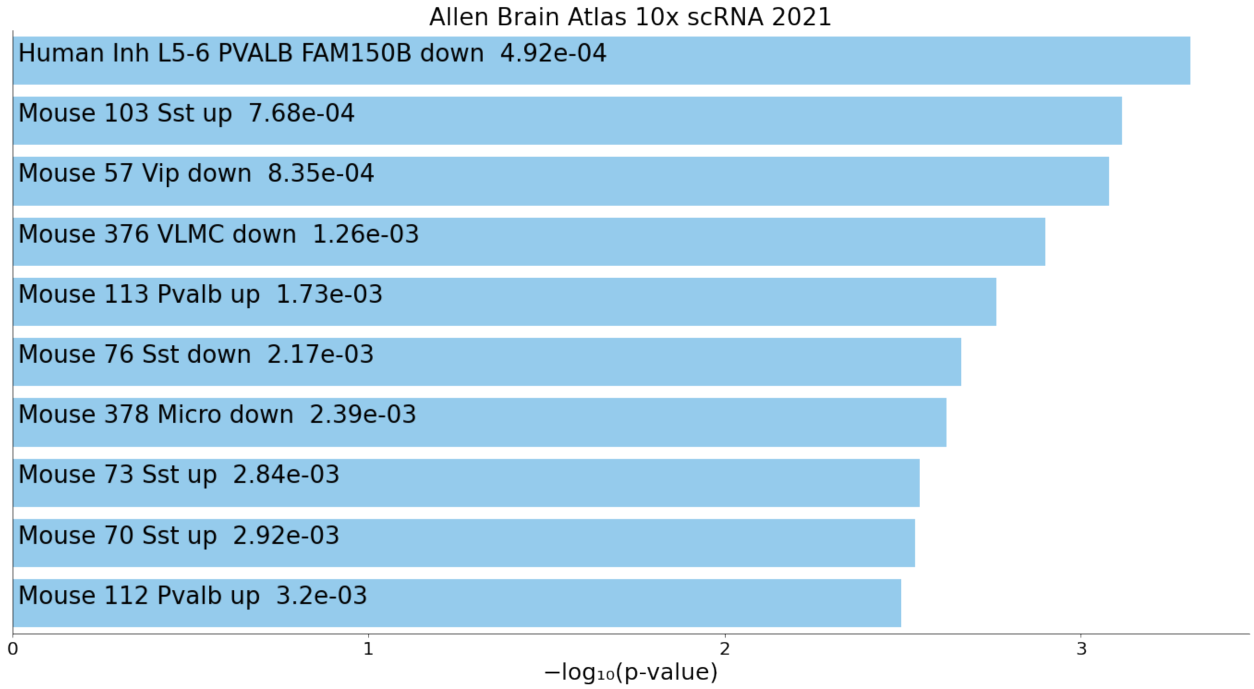

Figure S17 frontal_cortex_down2(brain up)

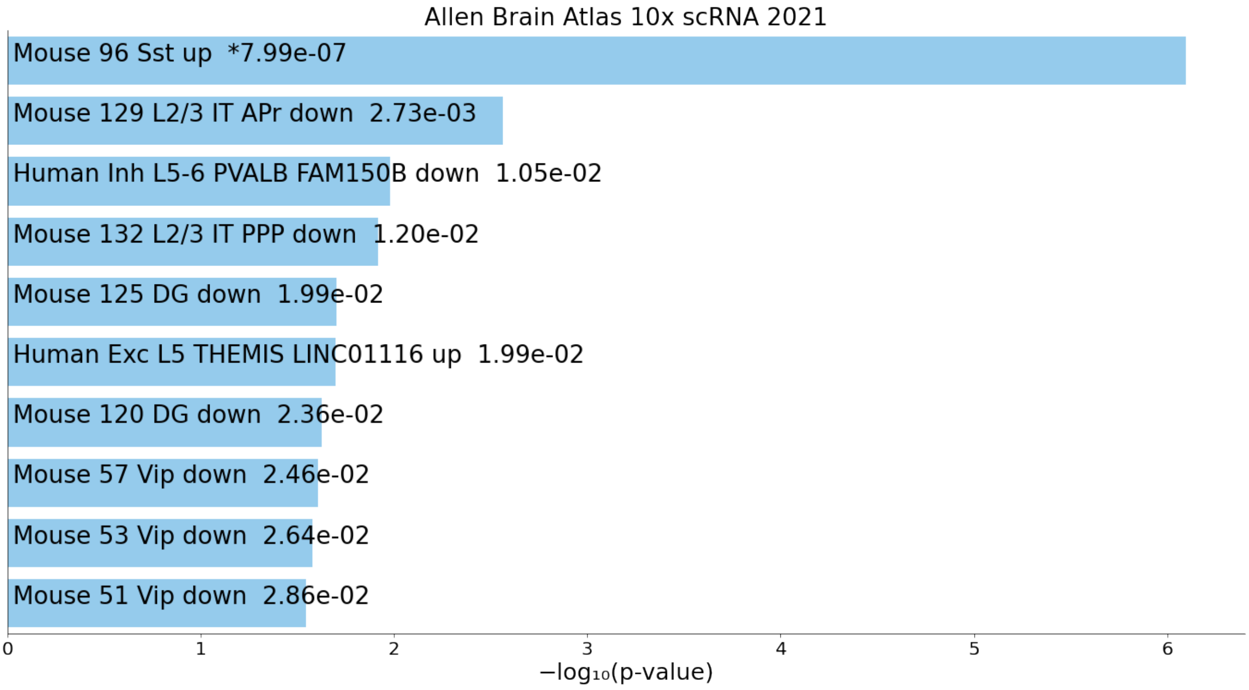

Figure S18 left_cerebral_cortex_up

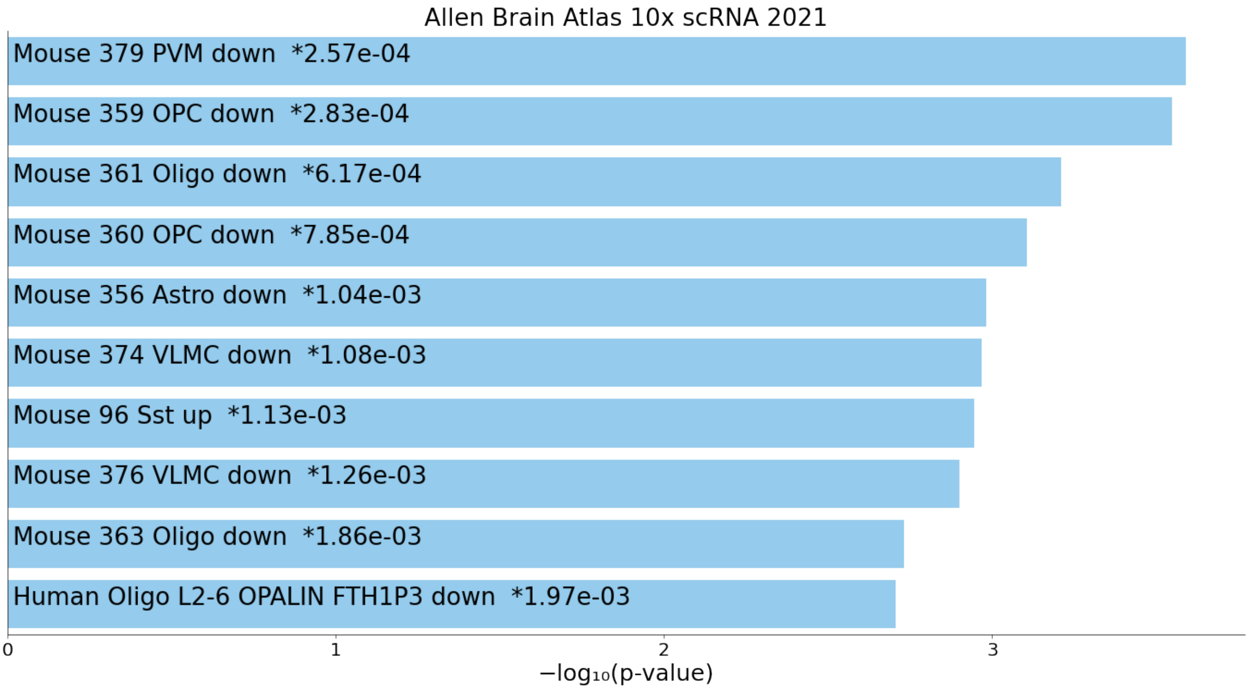

Figure S19 left_cerebral_cortex_down(brain up)

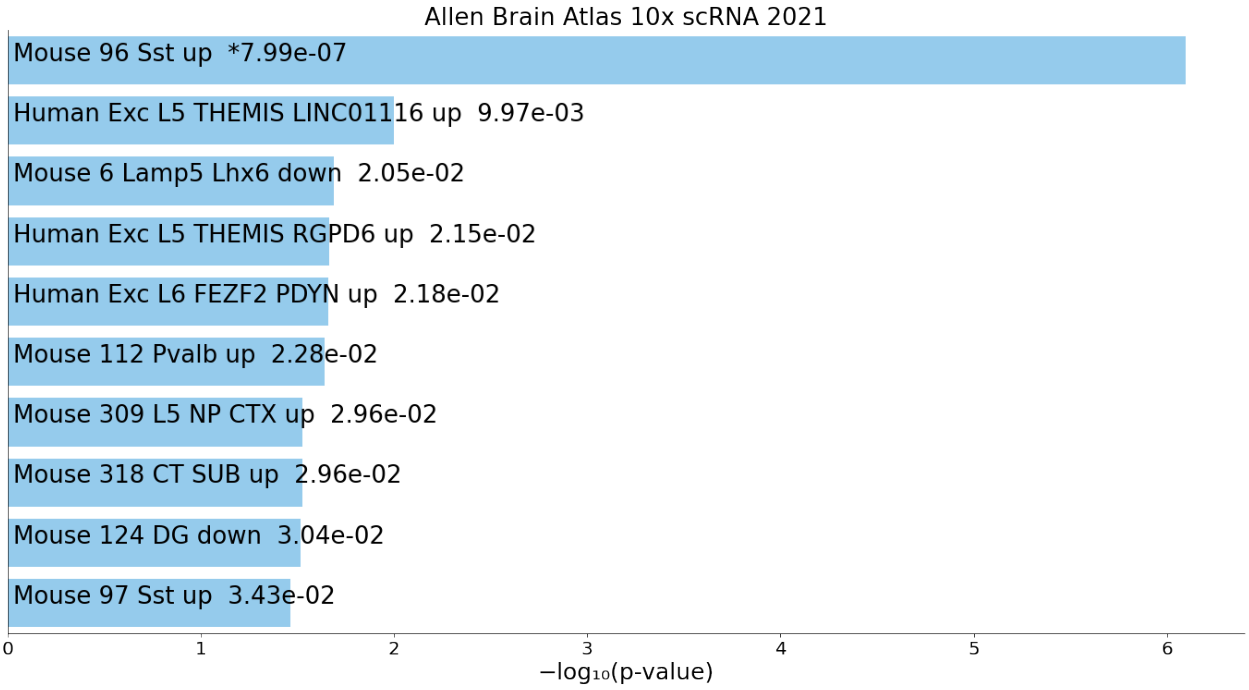

Figure S20 right_cerebral_cortex_up

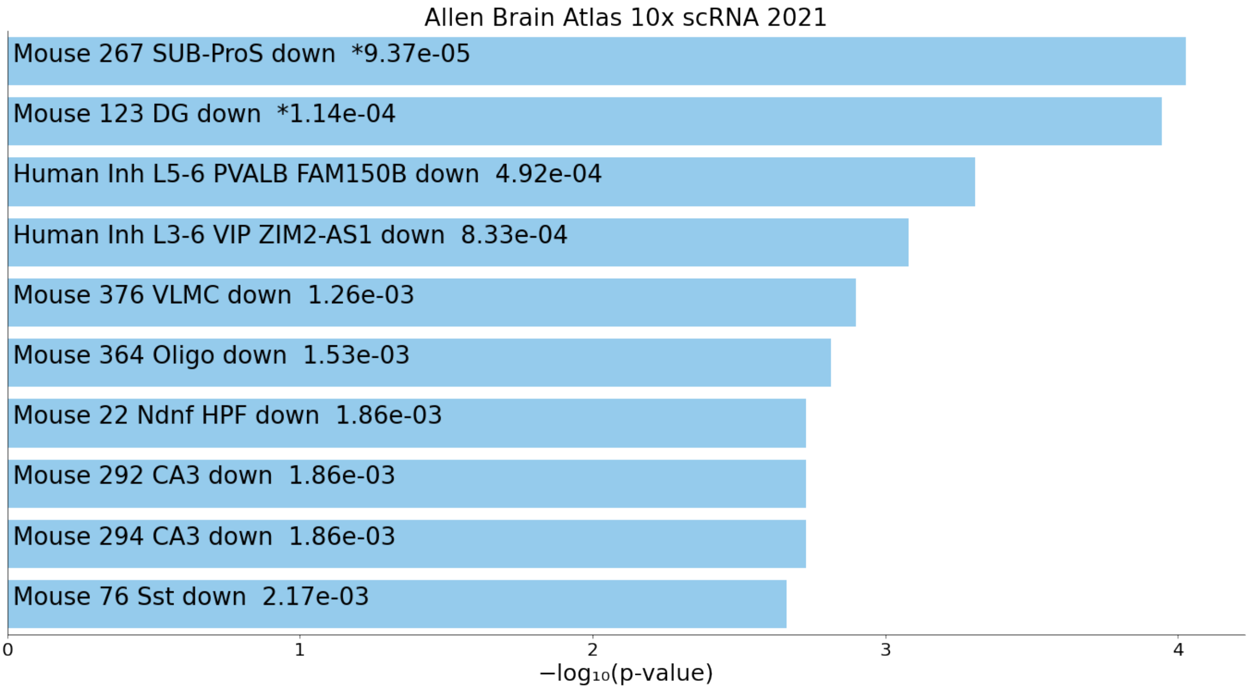

Figure S21 right_cerebral_cortex_down(brain up)

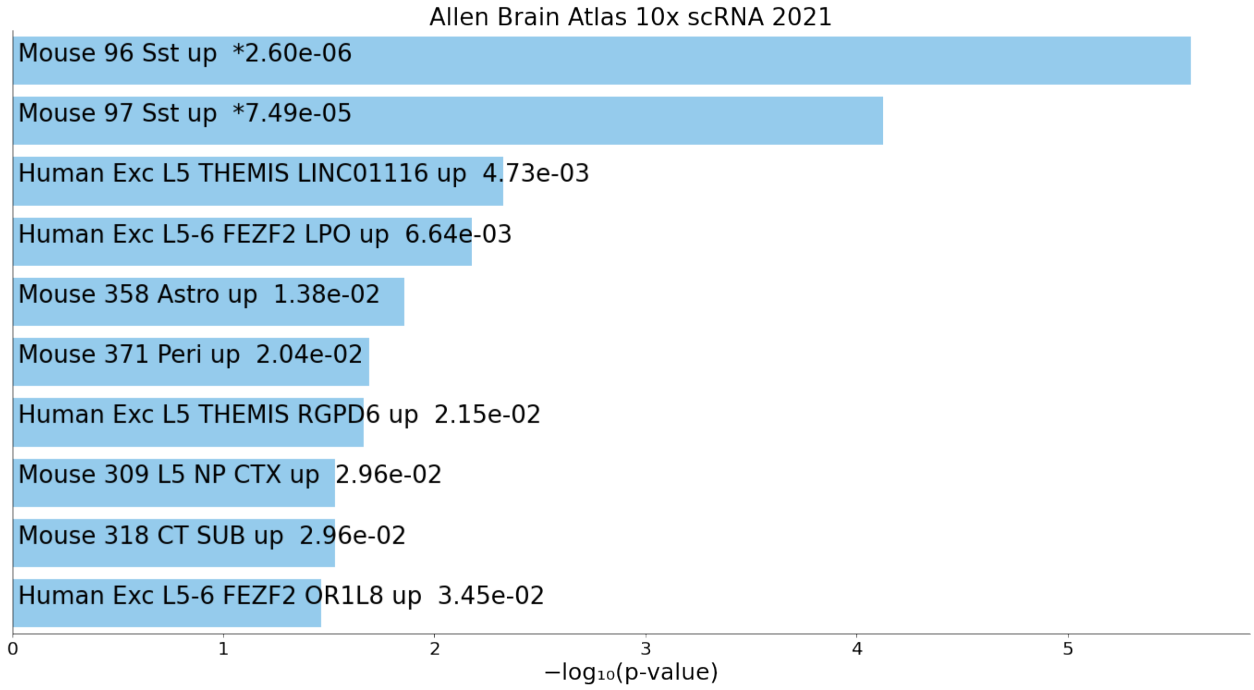

Figure S22 hippocampal_layer_up

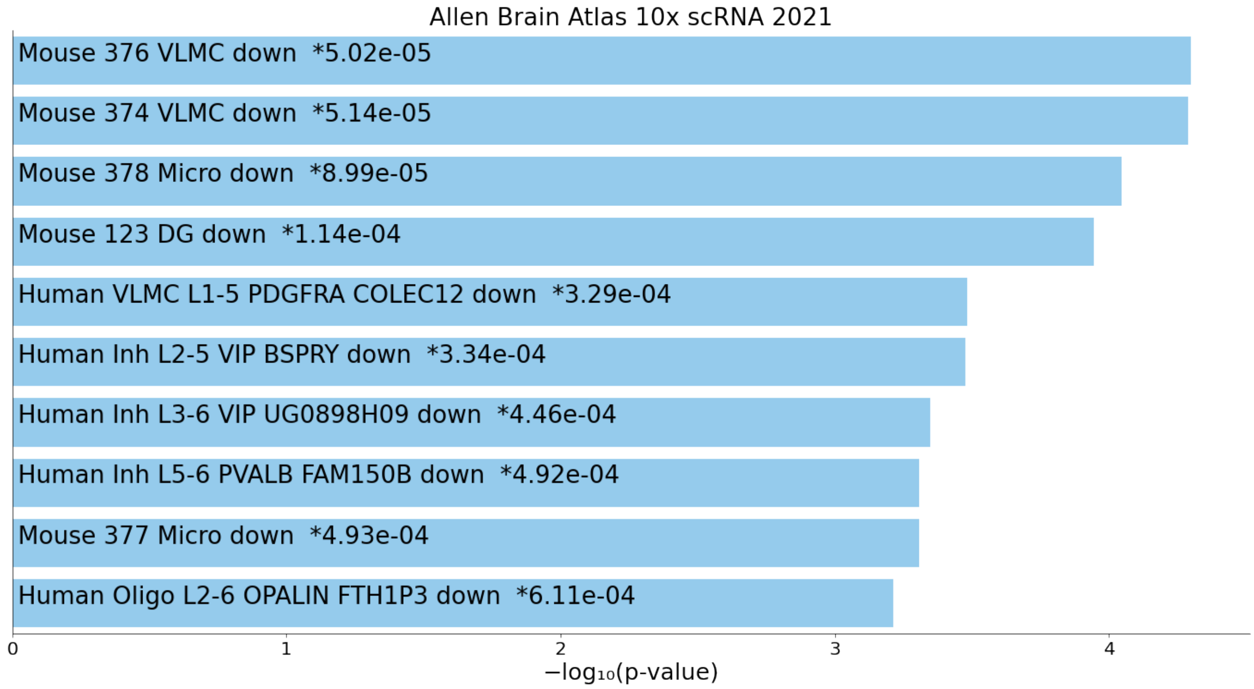

Figure S23 hippocampal_layer_down(brain up)

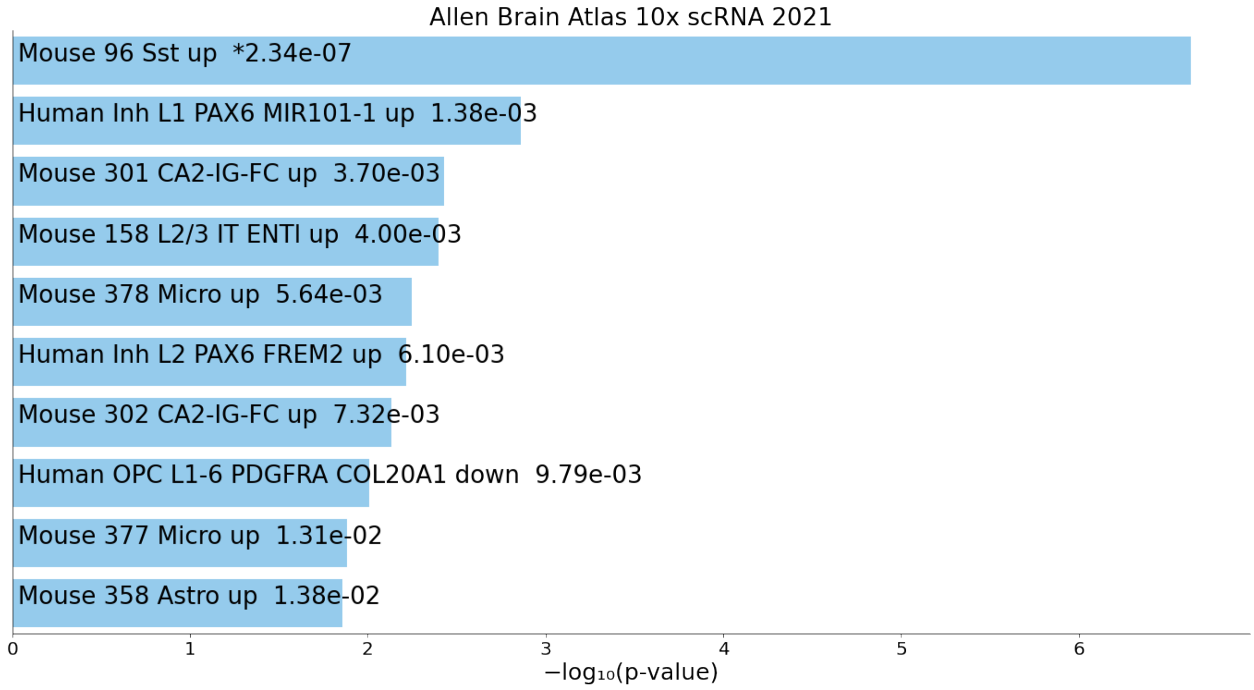

Figure S24 frontal_cortex_up

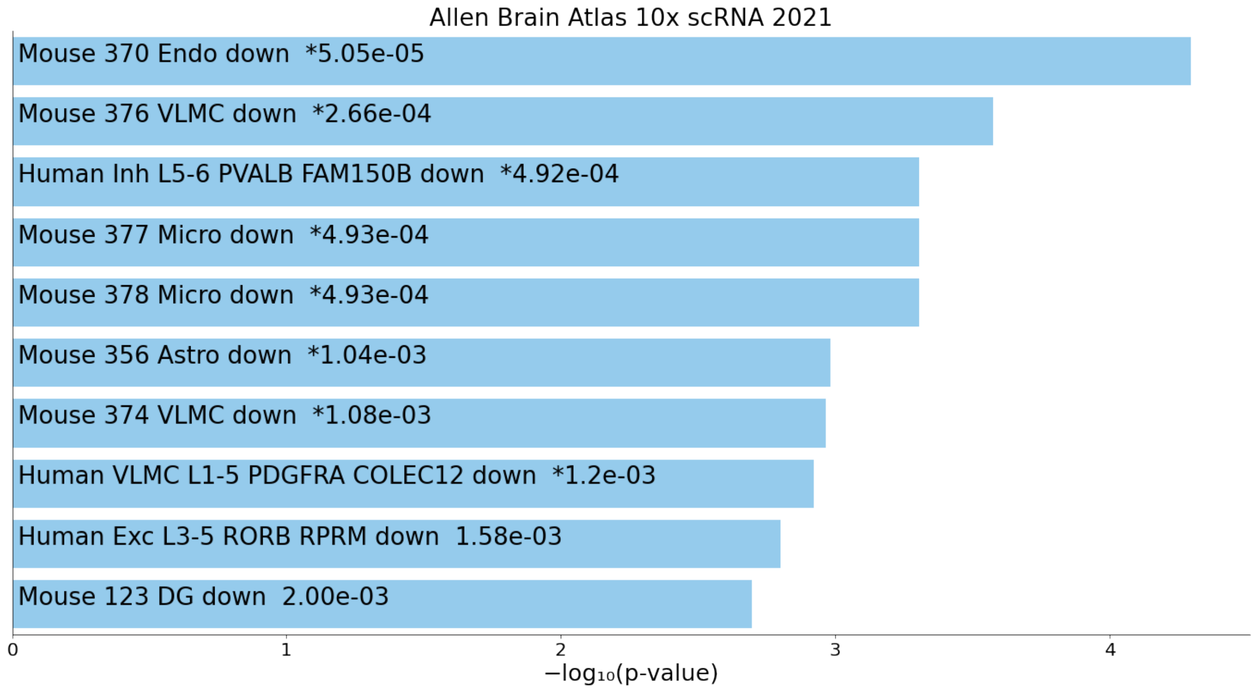

Figure S25 frontal_cortex_down(brain up)

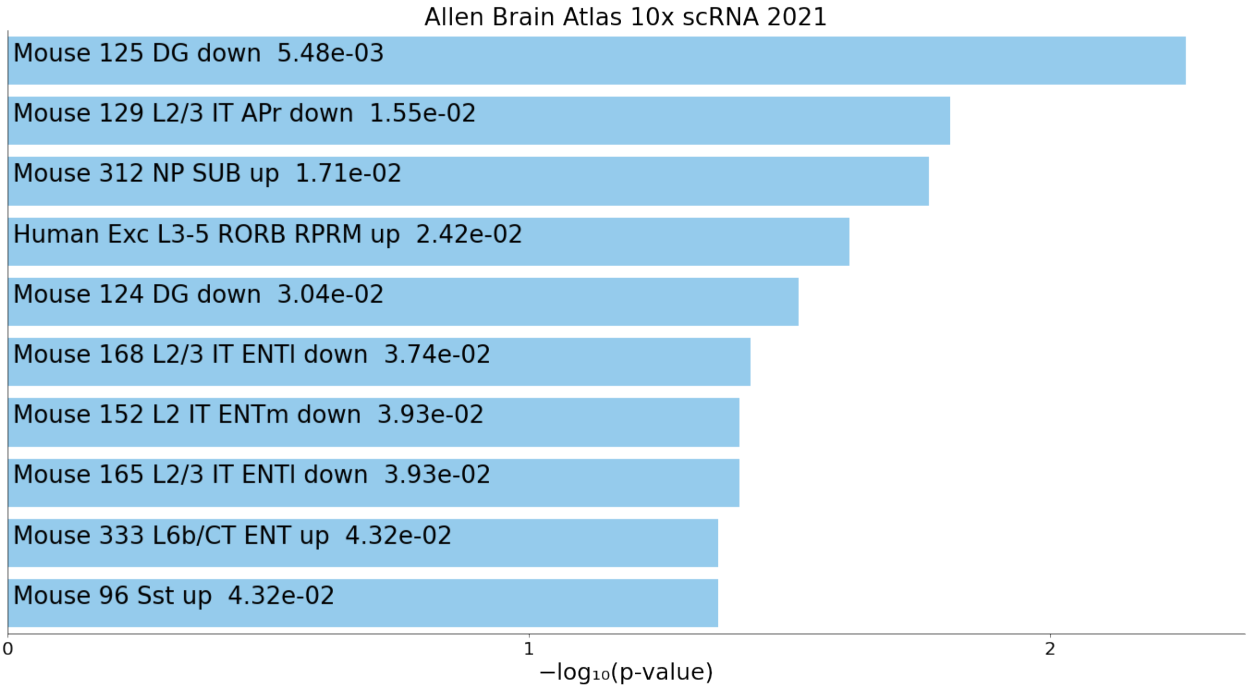

Figure S26 left_cerebral_cortex_up1

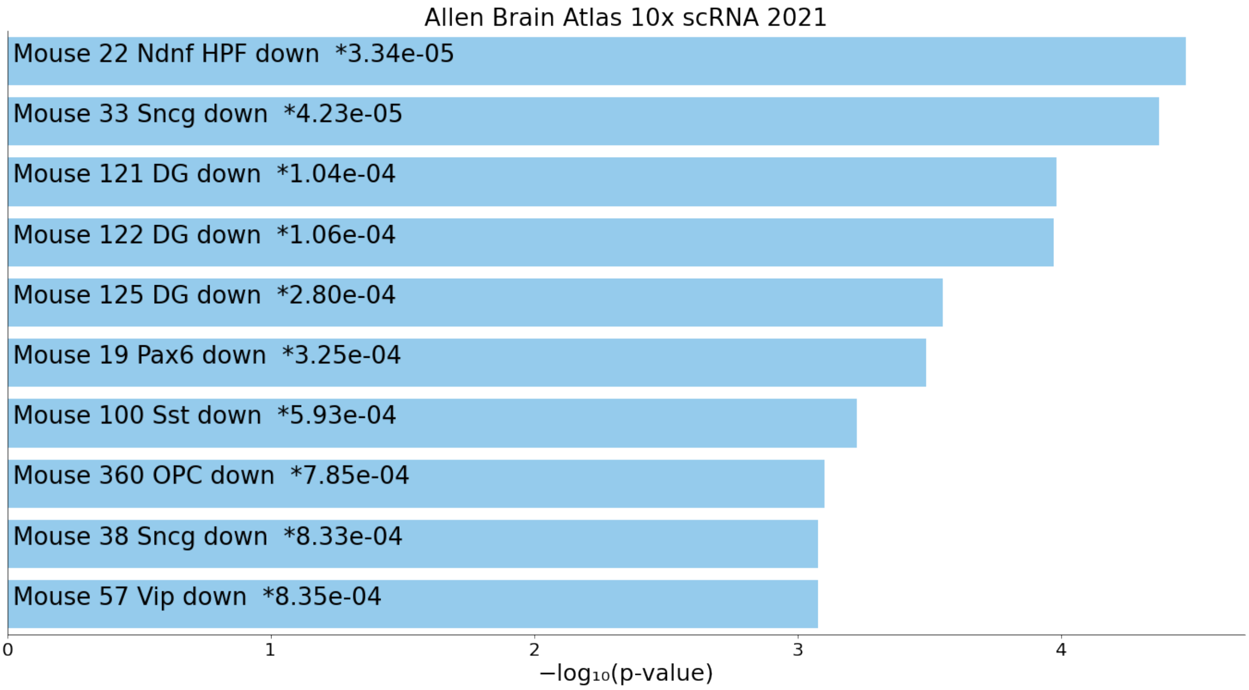

Figure S27 left_cerebral_cortex_down1(brain up)

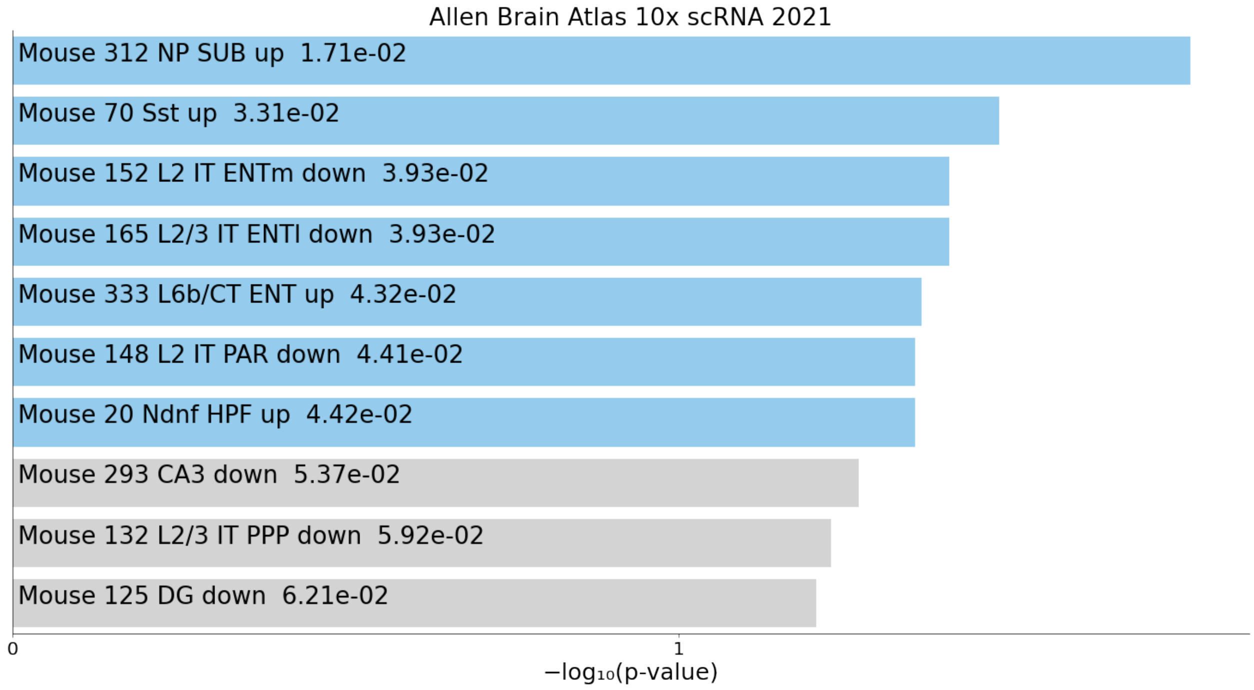

Figure S28 right_cerebral_cortex_up1

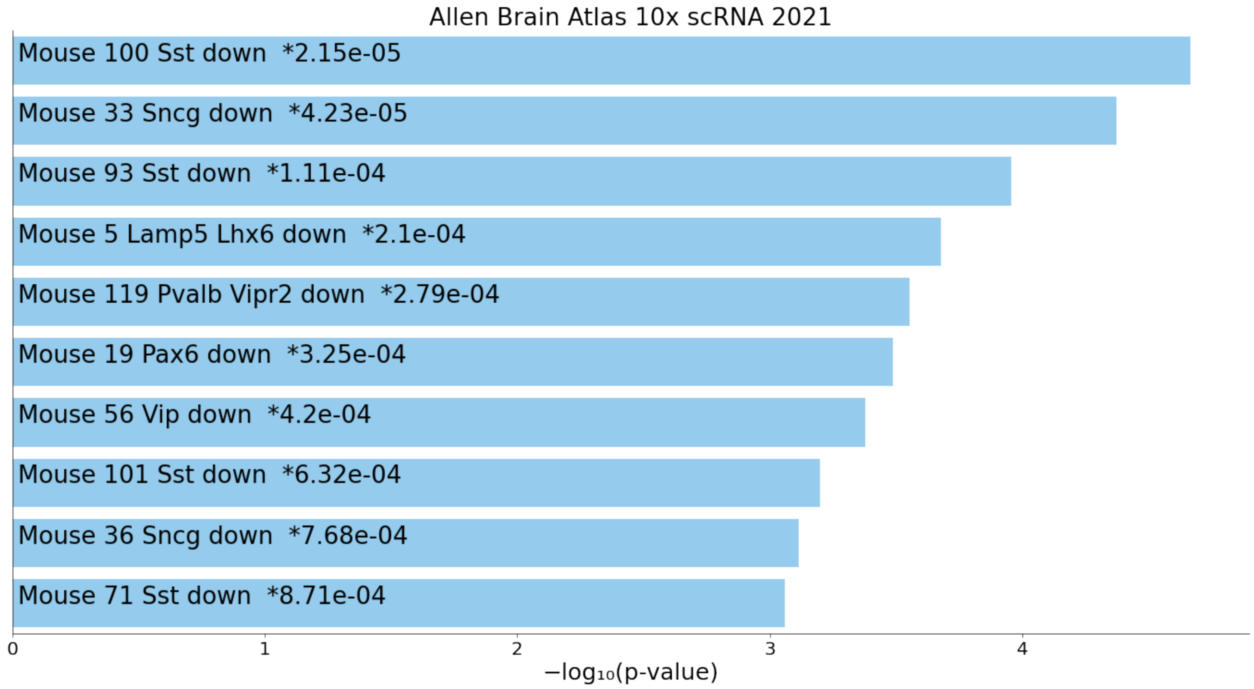

Figure S29 right_cerebral_cortex_down1(brain up)

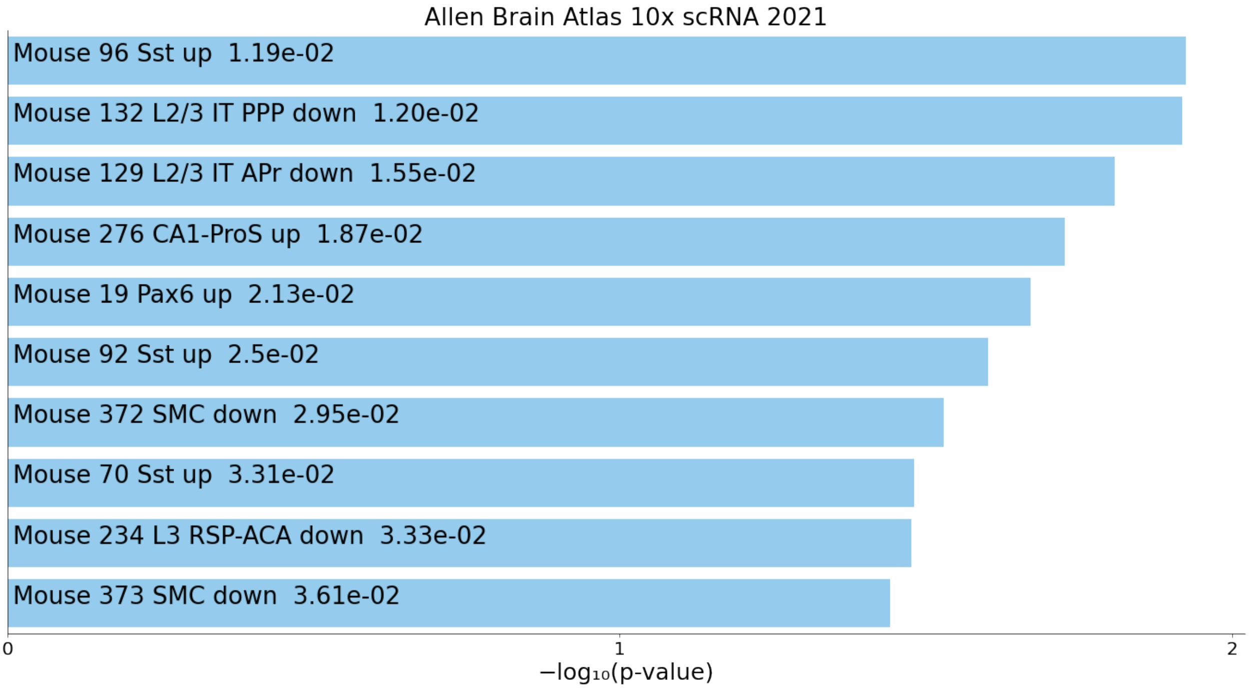

Figure S30 hippocampal_layer_up1

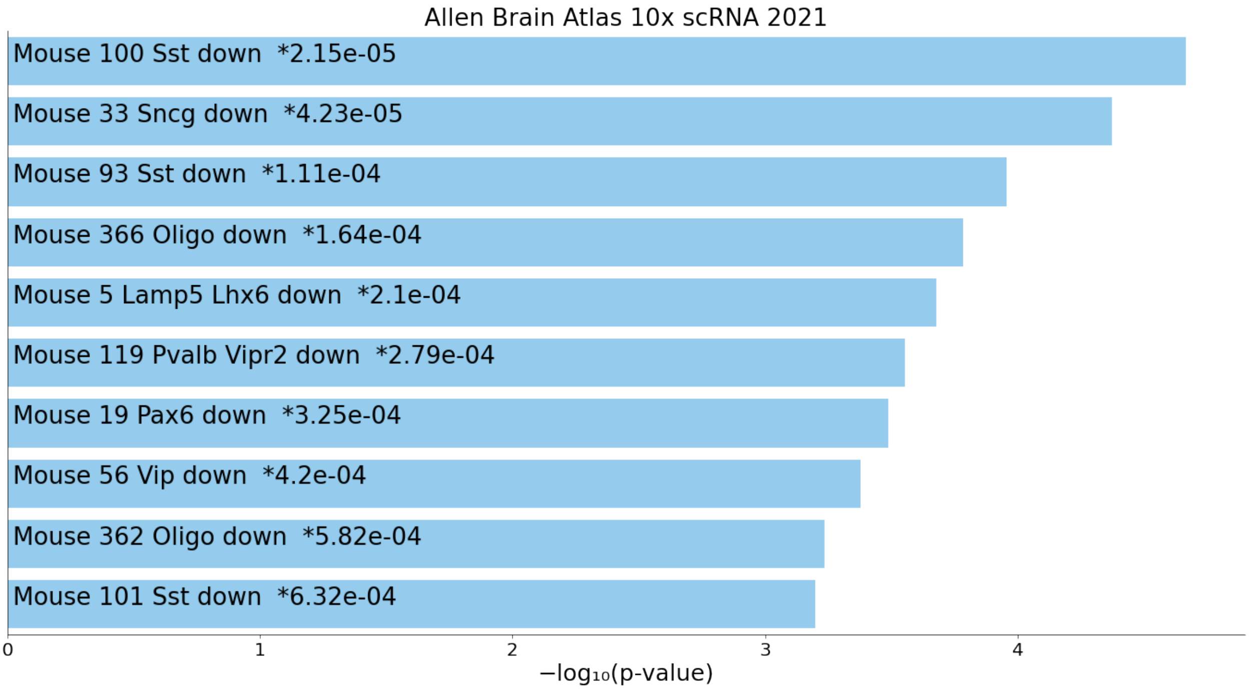

Figure S31 hippocampal_layer_down1(brain up)

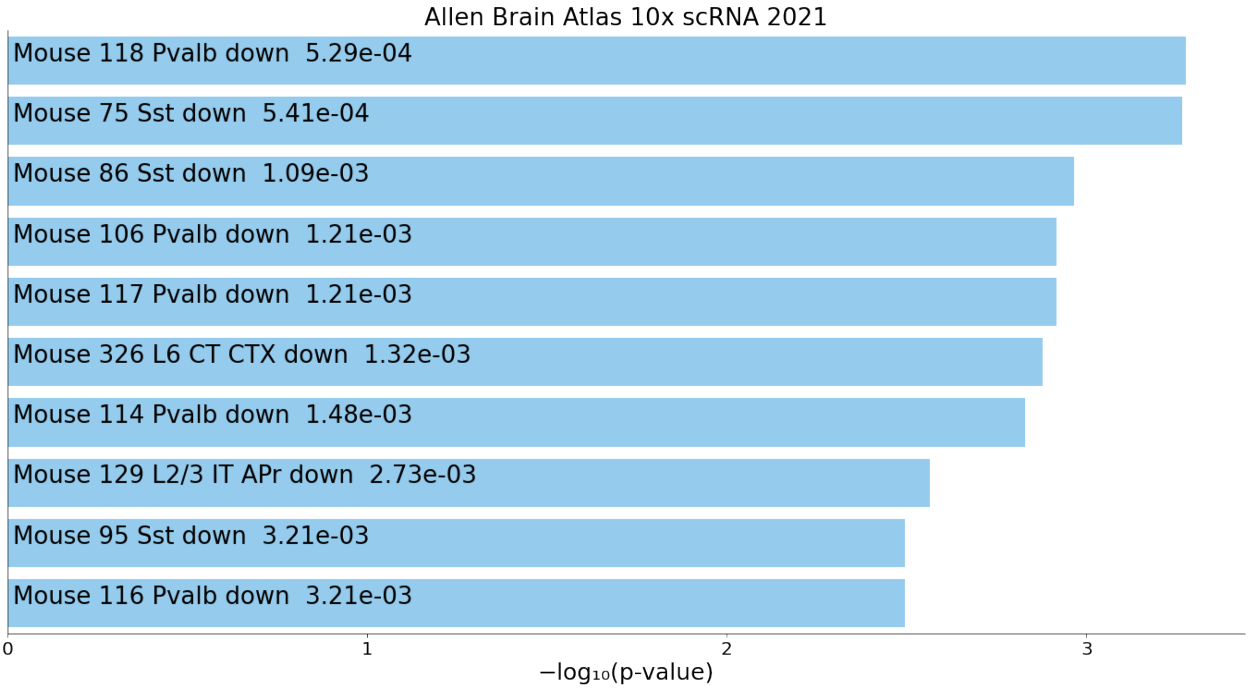

Figure S32 frontal_cortex_up1

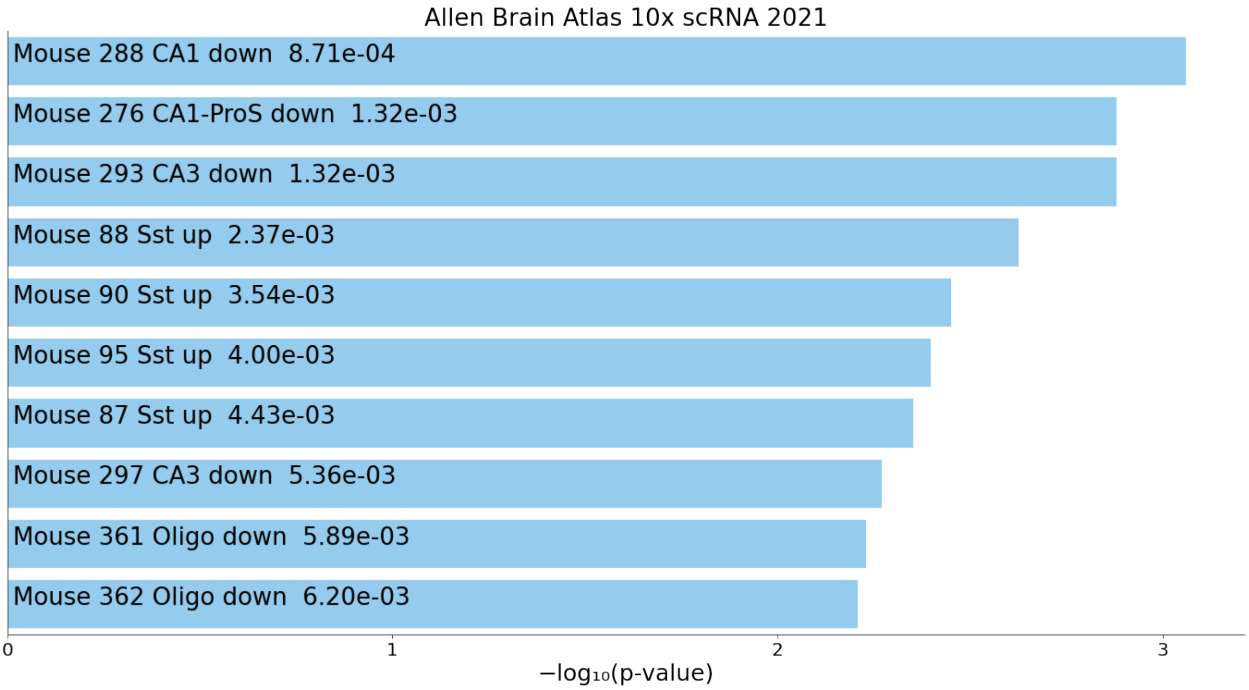

Figure S33 frontal_cortex_down1(brain up)

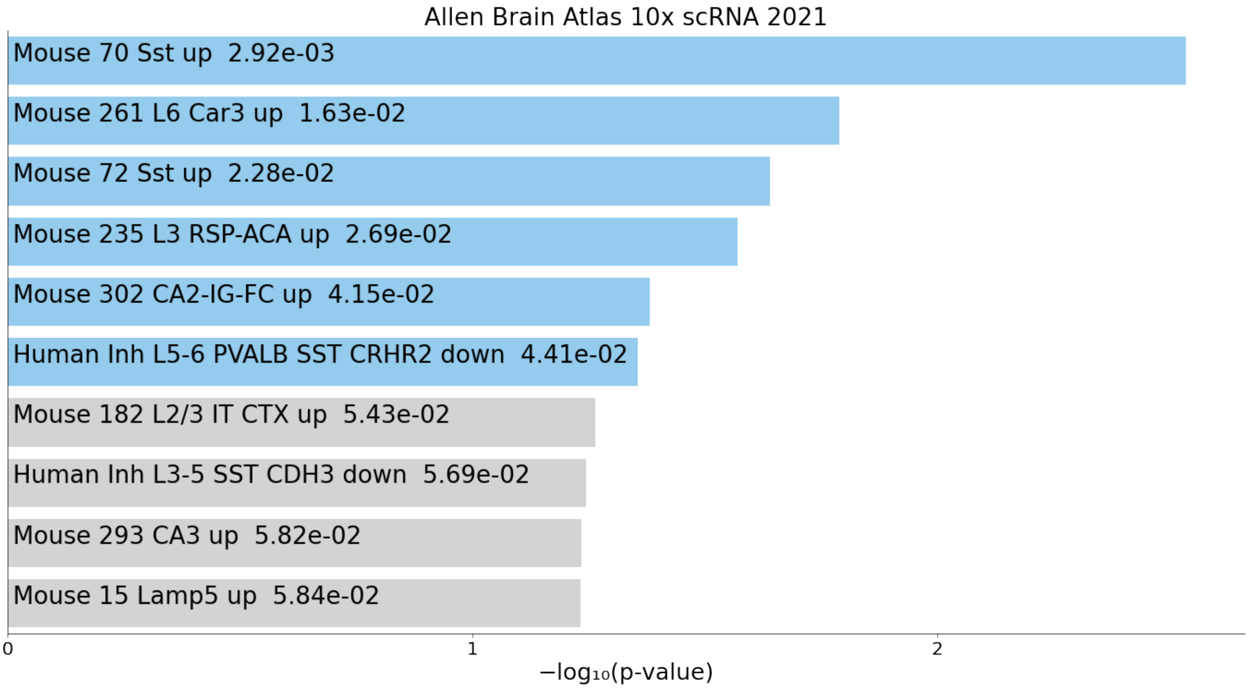

Figure S34 left_cerebral_cortex_up2

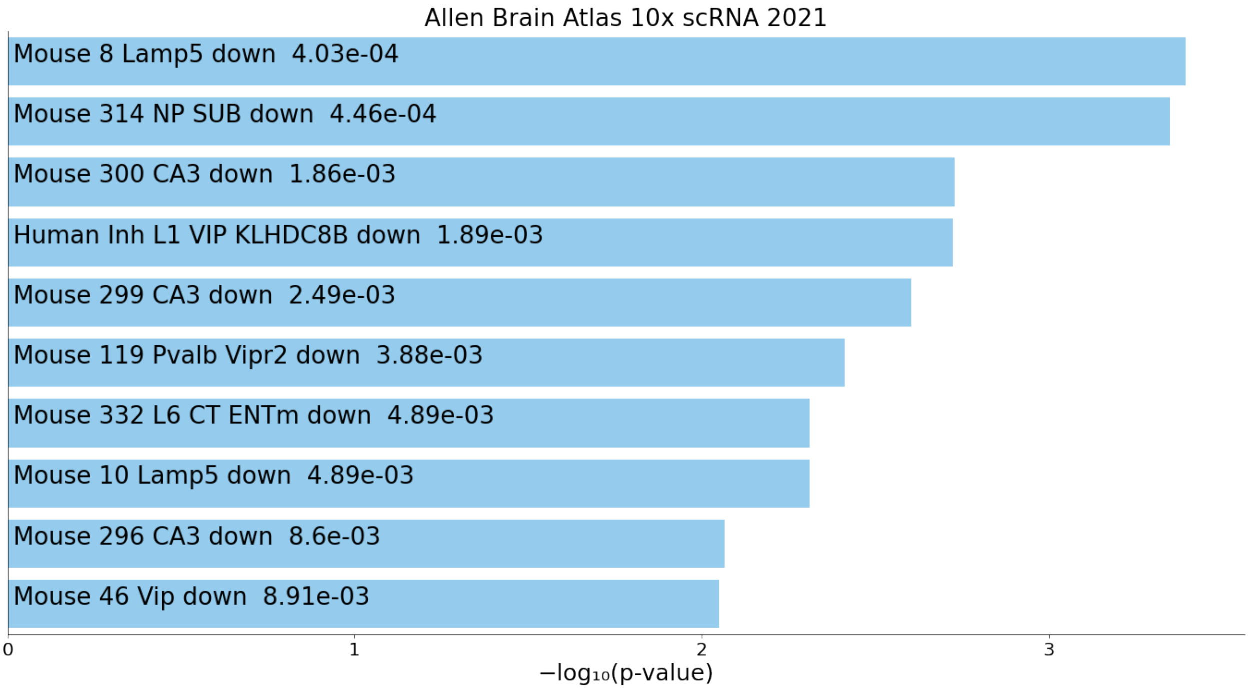

Figure S35 left_cerebral_cortex_down2(brain up)

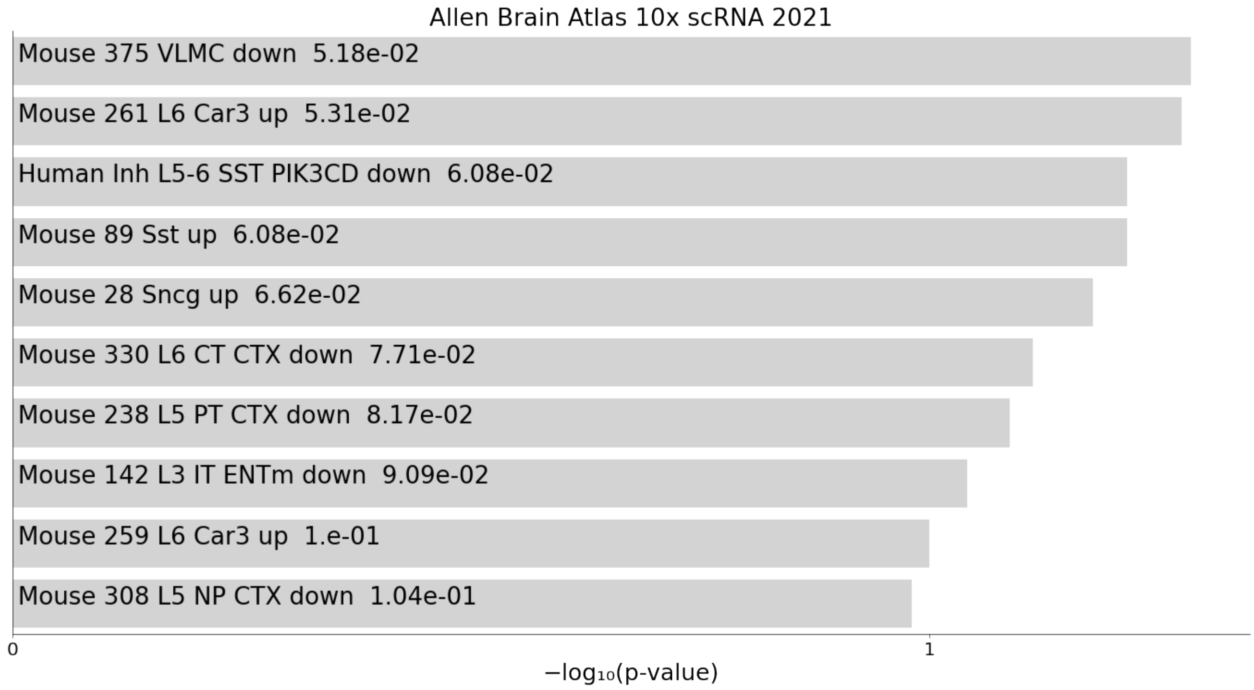

Figure S36 right_cerebral_cortex_up2

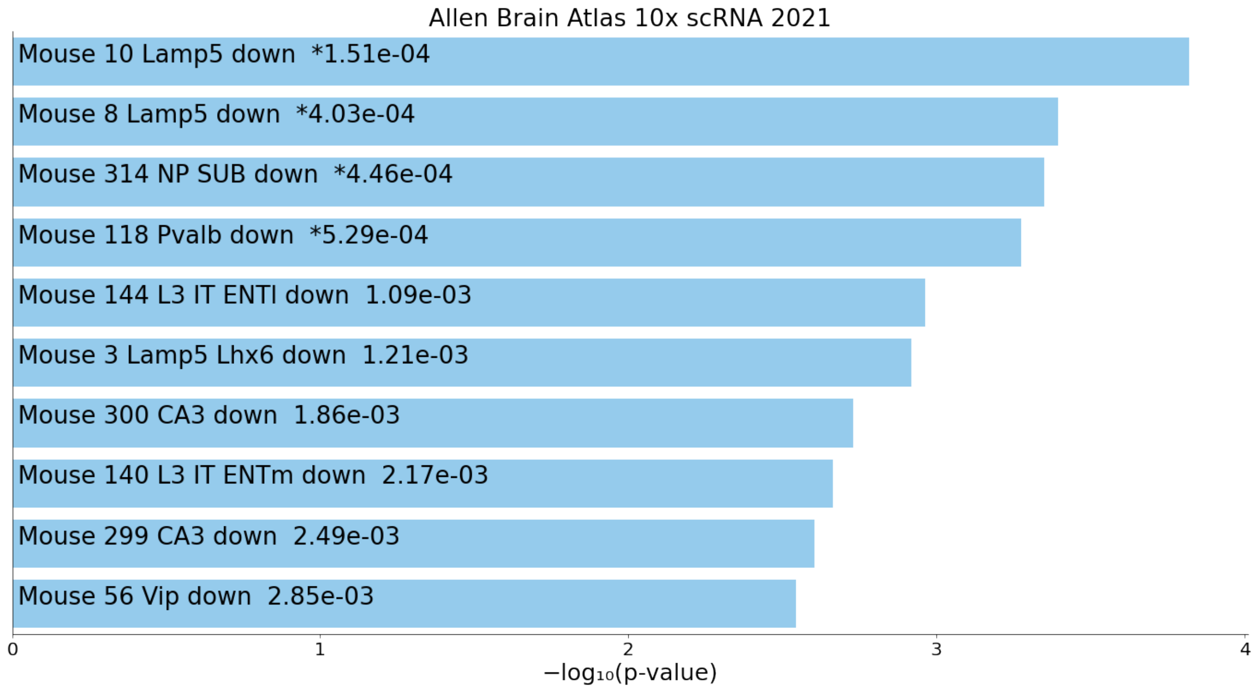

Figure S37 right_cerebral_cortex_down2(brain up)

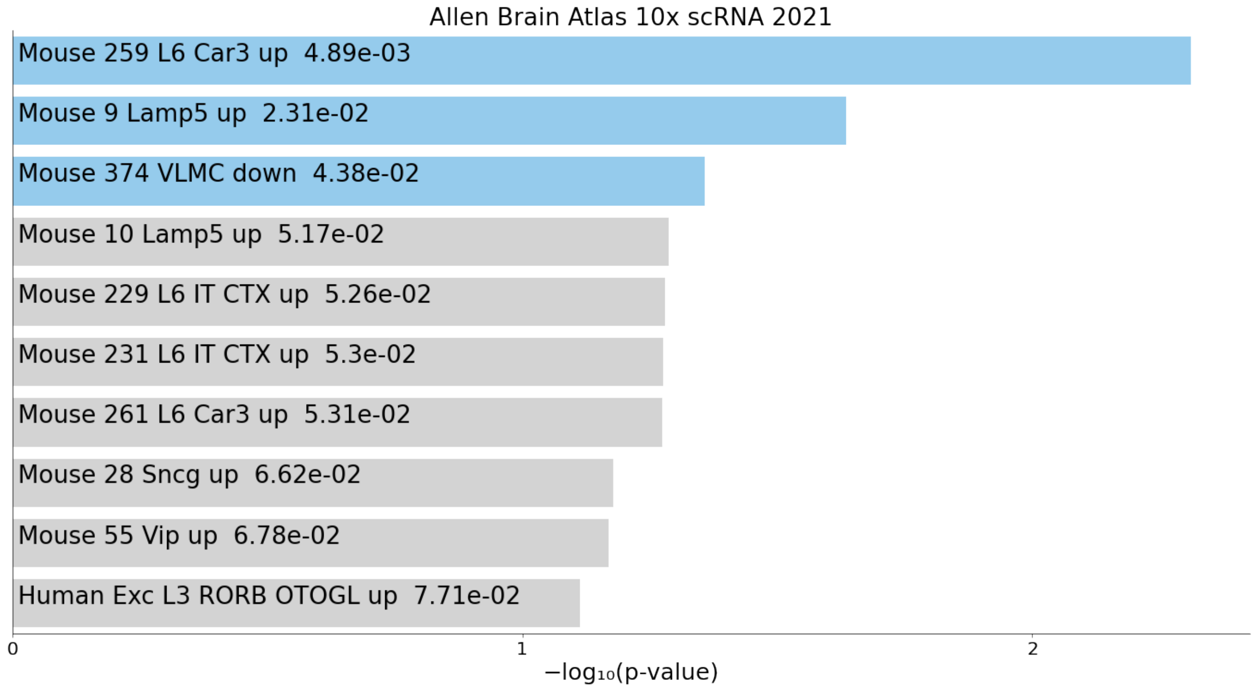

Figure S38 hippocampal_layer_up2

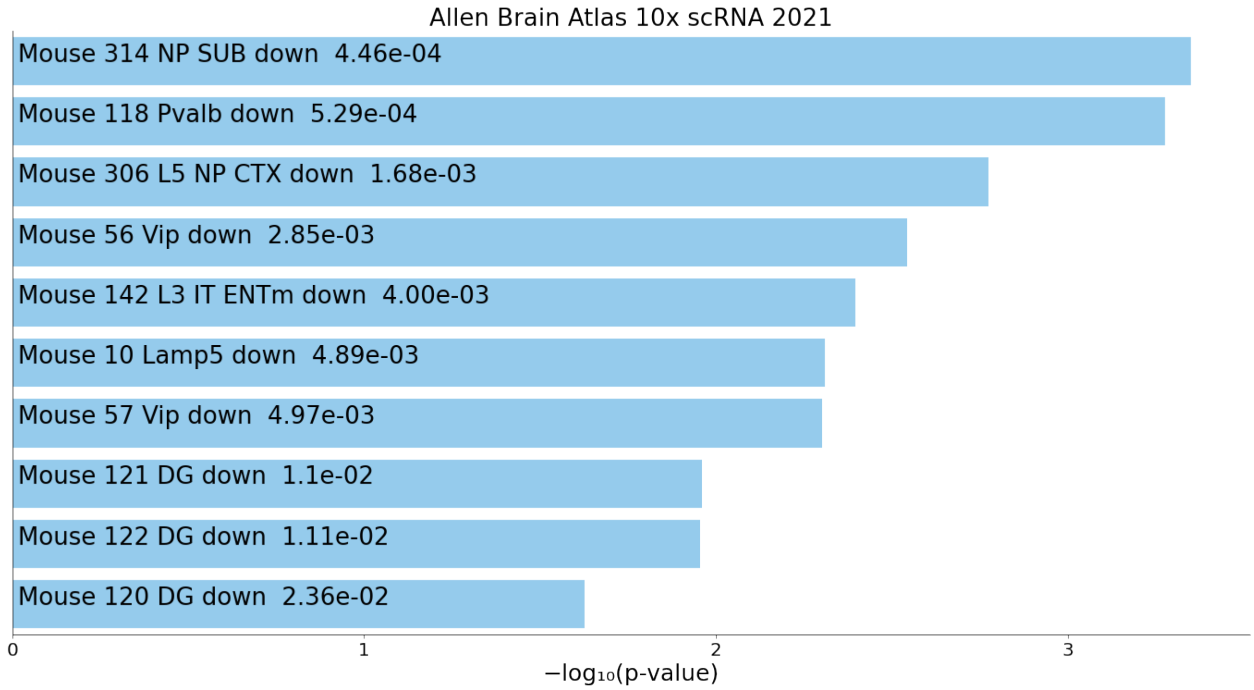

Figure S39 hippocampal_layer_down2(brain up)

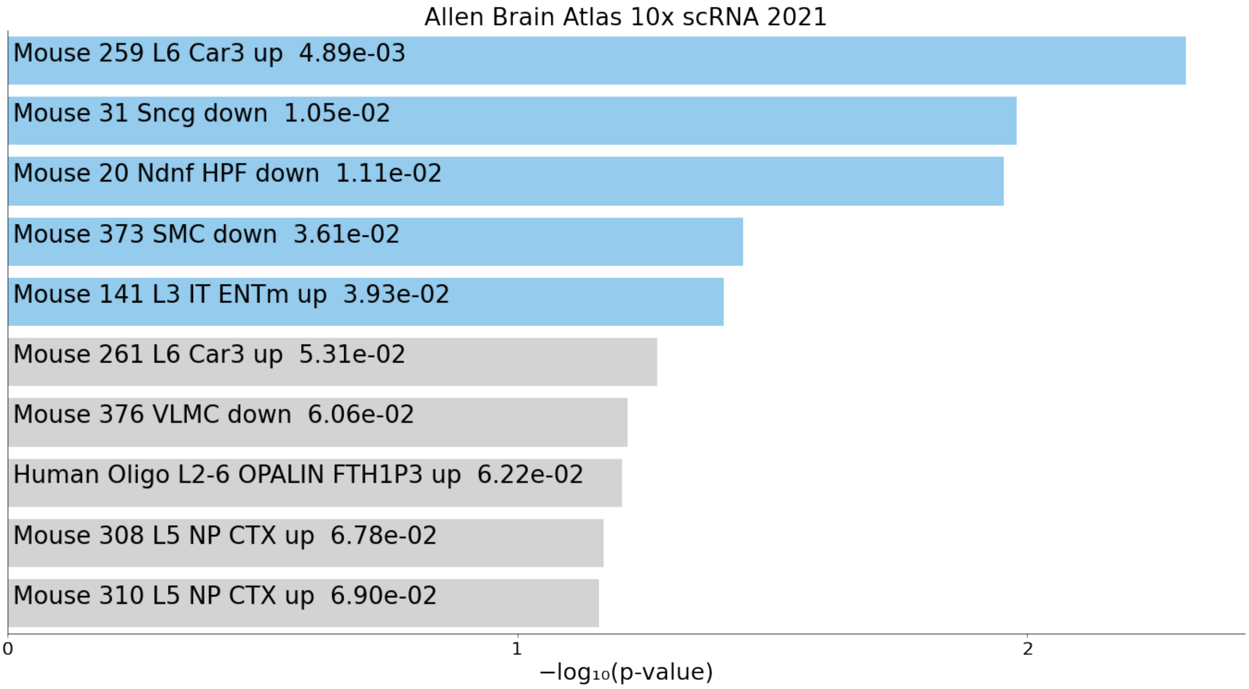

Figure S40 frontal_cortex_up2

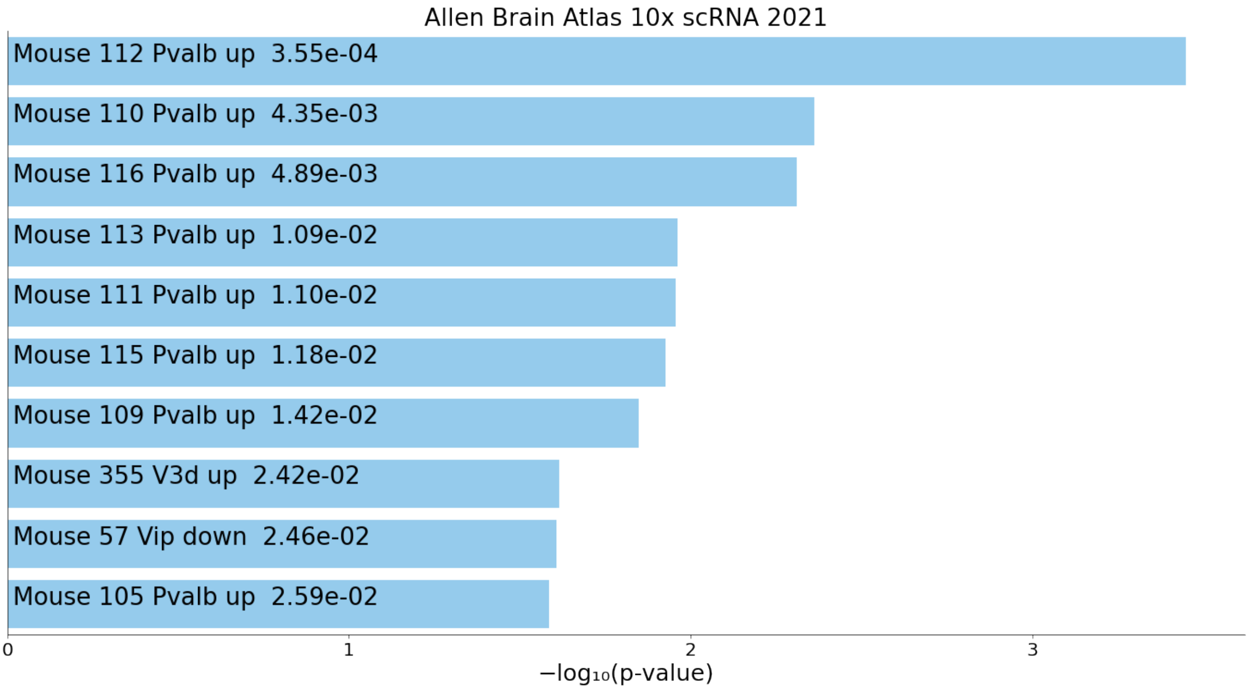

Figure S41 frontal_cortex_down2(brain up)
