# Supplementary material for: Somatostatin-Expressing Neurons Regulate Sleep Deprivation and Recovery
Source: Genes (Basel). 2026 Jan 1;17(1):51. doi: 10.3390/genes17010051 (PMC12840664; doi:10.3390/genes17010051)
Supplement: Supplementary file 1 [file genes-17-00051-s001.zip › Supplementary File S7-Figures_S42-S44.pdf]

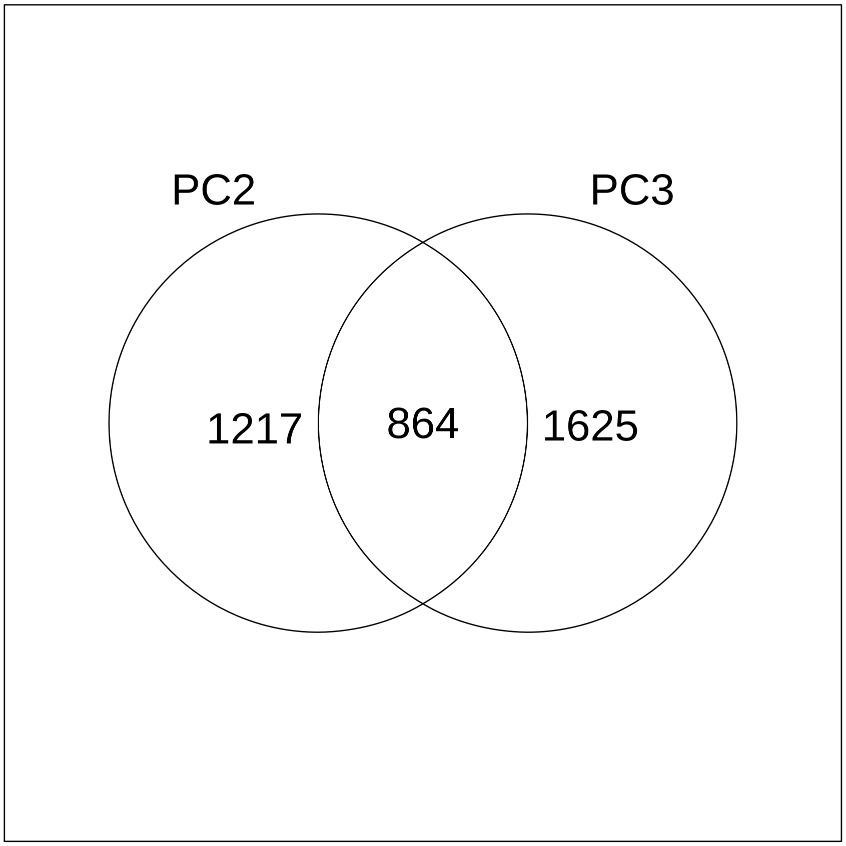

Figure S42: Venn diagram between gene symbols selected by $\ell_1 = 2$ and $\ell_1 = 3$, respectively. PC2: $\ell_1 = 2$ and PC3: $\ell_1 = 3$.

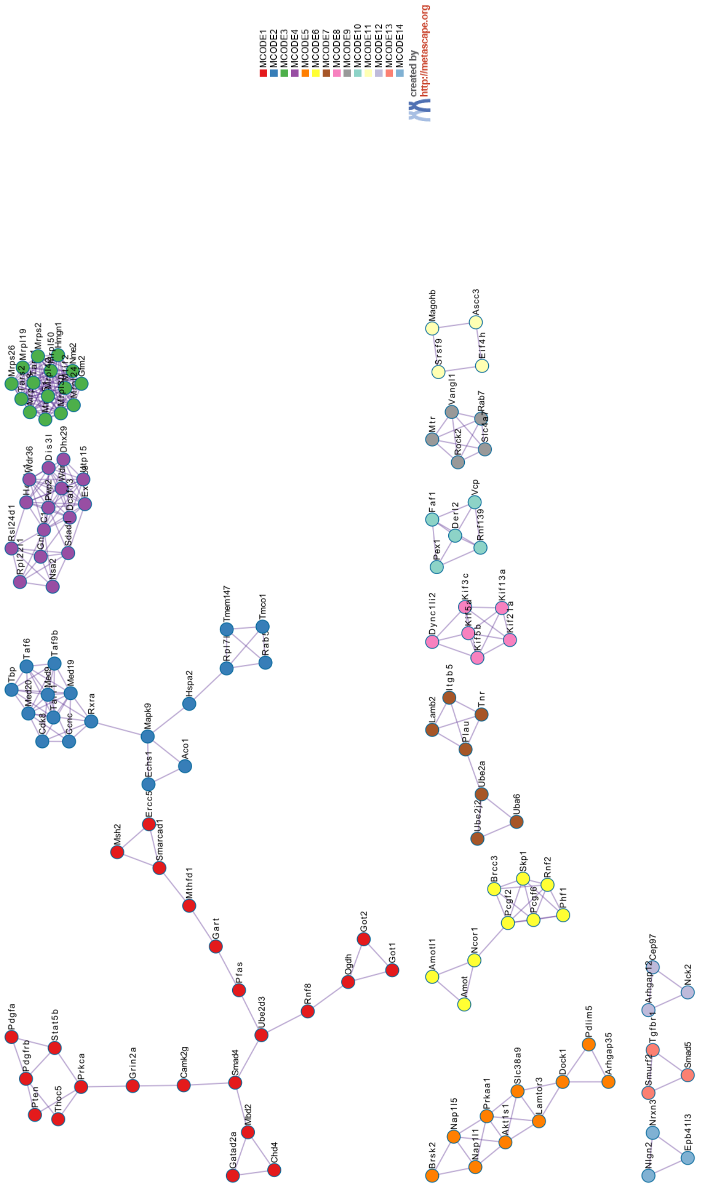

Figure S43: Protein–protein interaction network and MCODE components identified in the gene lists. For annotation of cluster, see Tables ?? and ??.

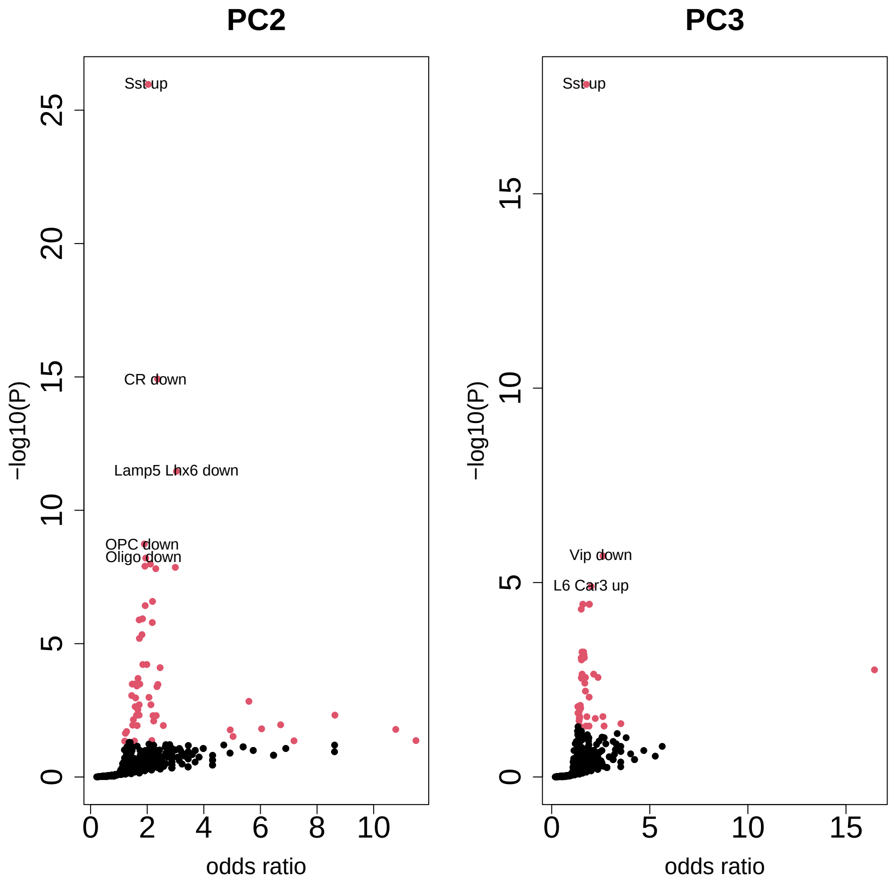

Figure S44: Volcano plot of cell clusters in "Allen Brain Atlas 10x scRNA 2021" category. Red ones are associated with adjusted P-values less than 0.05. PC2: $\ell_1 = 2$ and PC3: $\ell_1 = 3$.
