# Supplementary figures and images for: Somatostatin-Expressing Neurons Regulate Sleep Deprivation and Recovery
Source: Genes (Basel). 2026 Jan 1;17(1):51. doi: 10.3390/genes17010051 (PMC12840664; doi:10.3390/genes17010051)

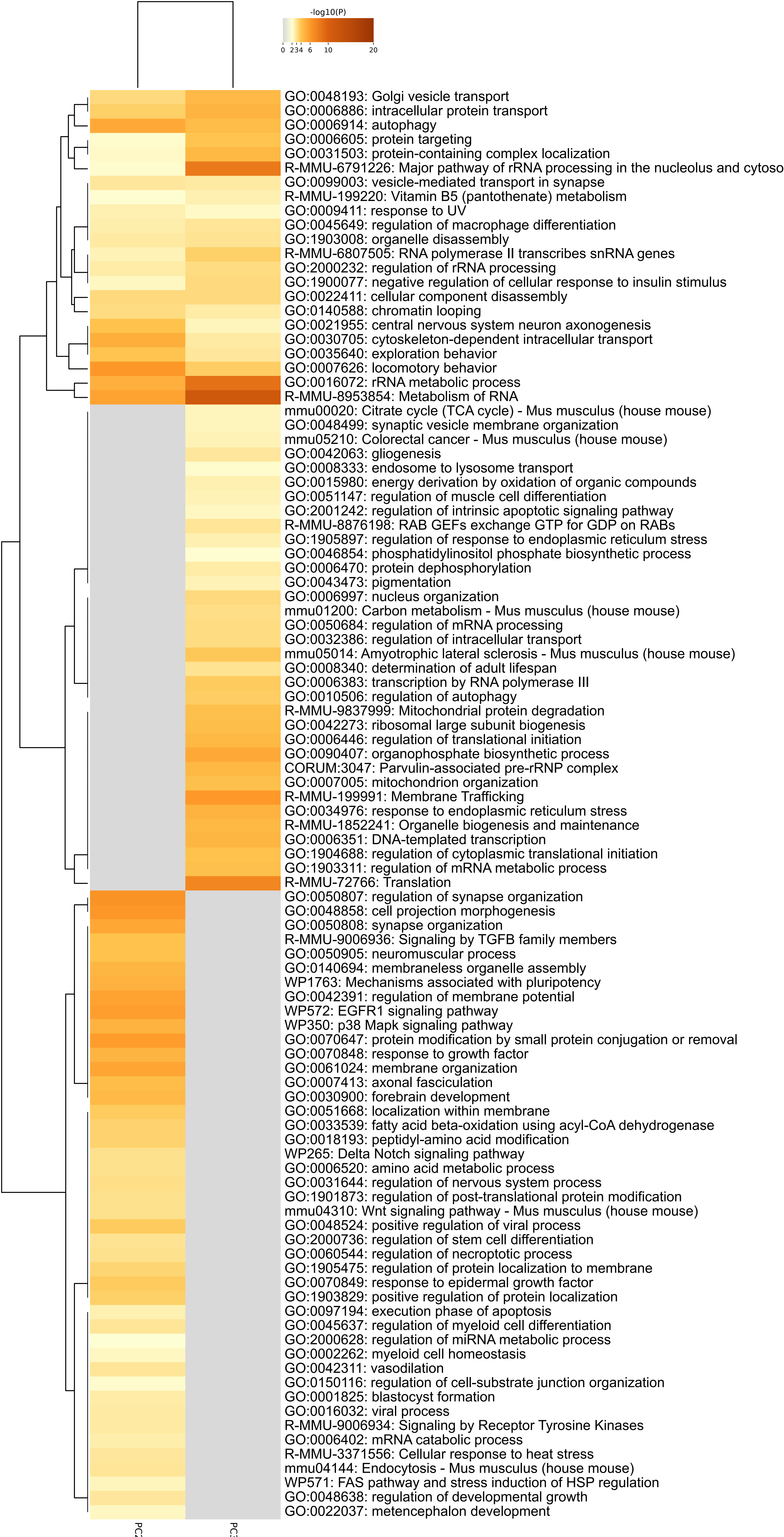

Supplement: Supplementary file 1 [file genes-17-00051-s001.zip › Supplementary File S1-Figure_S1.pdf]
