# Supplementary material for: Somatostatin-Expressing Neurons Regulate Sleep Deprivation and Recovery
Source: Genes (Basel). 2026 Jan 1;17(1):51. doi: 10.3390/genes17010051 (PMC12840664; doi:10.3390/genes17010051)
Supplement: Supplementary file 1 [file genes-17-00051-s001.zip › Supplementary File S2-Metascape.zip!/Metascape/all.tzpz10j5i/Enrichment_GO/ColorByCluster.pdf]

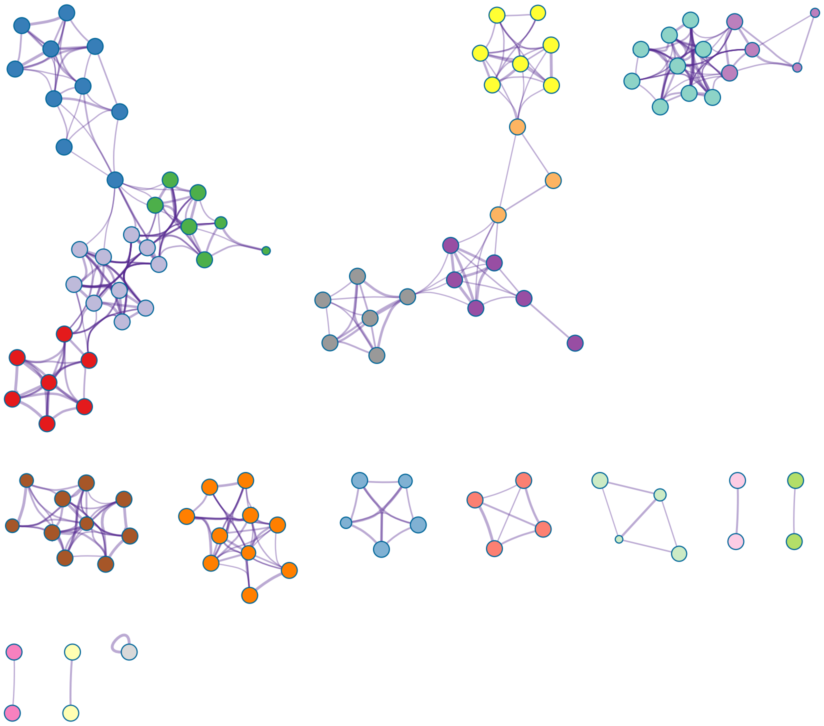

Translation
Metabolism of RNA
rRNA metabolic process
membrane organization
DNA-templated transcription
Membrane Trafficking
cytoskeleton-dependent intracellular transport
intracellular protein transport
autophagy
cell projection morphogenesis
cellular component disassembly
Major pathway of rRNA processing in the nucleolus a
response to growth factor
locomotory behavior
Golgi vesicle transport
positive regulation of protein localization
response to endoplasmic reticulum stress
Mitochondrial protein degradation
central nervous system neuron axonogenesis
RNA polymerase II transcribes snRNA genes

created by
http://metascape.org
