# Supplementary material for: Somatostatin-Expressing Neurons Regulate Sleep Deprivation and Recovery
Source: Genes (Basel). 2026 Jan 1;17(1):51. doi: 10.3390/genes17010051 (PMC12840664; doi:10.3390/genes17010051)
Supplement: Supplementary file 1 [file genes-17-00051-s001.zip › Supplementary File S2-Metascape.zip!/Metascape/all.tzpz10j5i/Enrichment_GO/ColorByCounts.pdf]

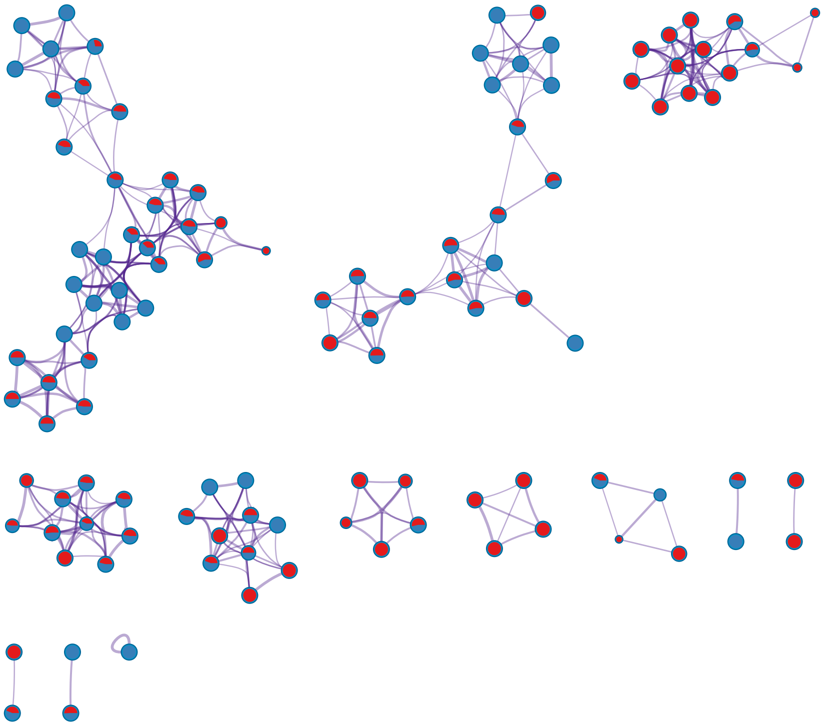

PC2
PC3

created by
http://metascape.org
