# Supplementary material for: Somatostatin-Expressing Neurons Regulate Sleep Deprivation and Recovery
Source: Genes (Basel). 2026 Jan 1;17(1):51. doi: 10.3390/genes17010051 (PMC12840664; doi:10.3390/genes17010051)
Supplement: Supplementary file 1 [file genes-17-00051-s001.zip › Supplementary File S2-Metascape.zip!/Metascape/all.tzpz10j5i/Enrichment_heatmap/HeatmapSelectedGO.pdf]

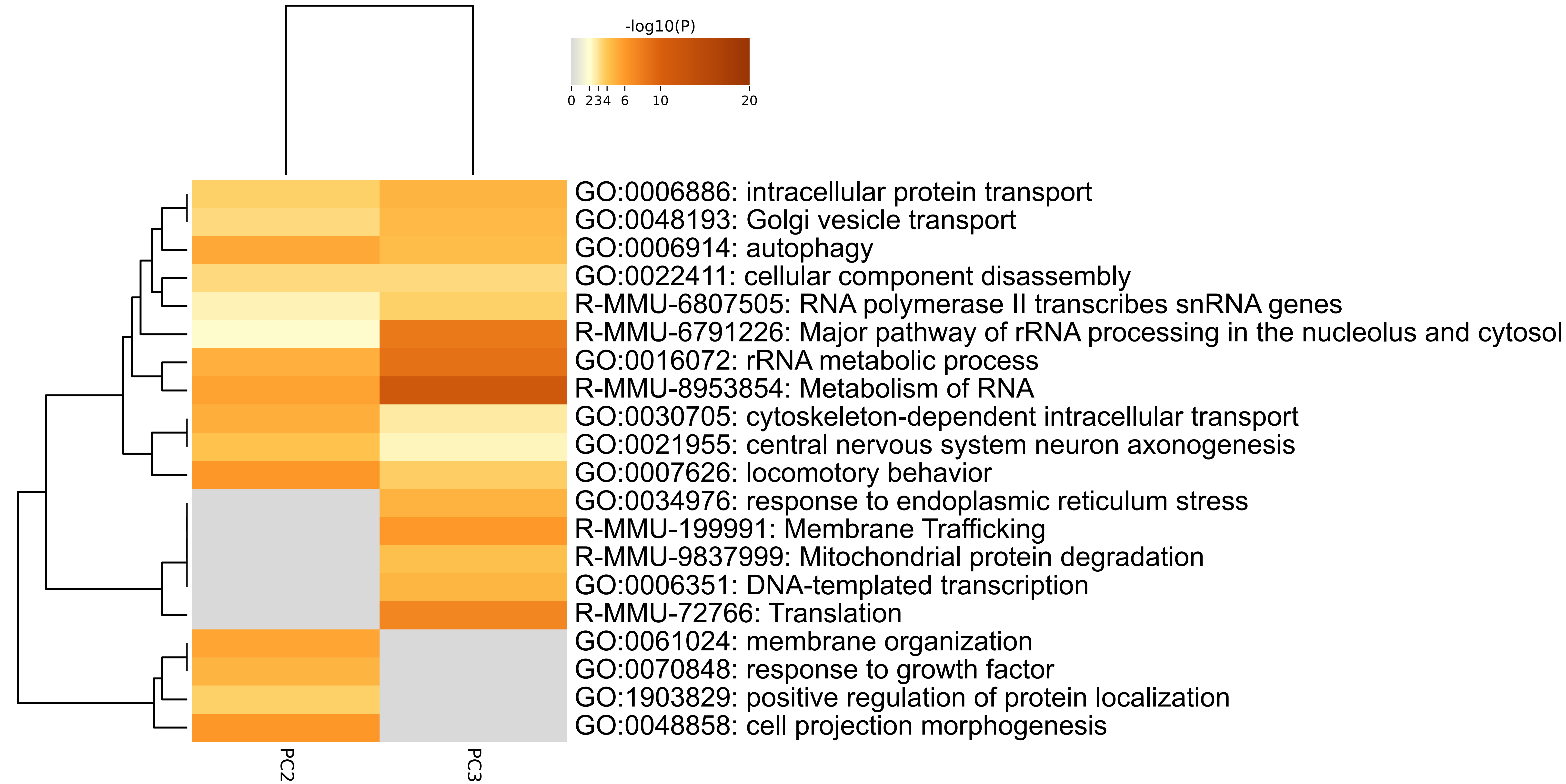

-log10(P)
0
2
3
4
6
10
20
GO:0006886: intracellular protein transport
GO:0048193: Golgi vesicle transport
GO:0006914: autophagy
GO:0022411: cellular component disassembly
R-MMU-6807505: RNA polymerase II transcribes snRNA genes
R-MMU-6791226: Major pathway of rRNA processing in the nucleolus and cytosol
GO:0016072: rRNA metabolic process
R-MMU-8953854: Metabolism of RNA
GO:0030705: cytoskeleton-dependent intracellular transport
GO:0021955: central nervous system neuron axonogenesis
GO:0007626: locomotory behavior
GO:0034976: response to endoplasmic reticulum stress
R-MMU-199991: Membrane Trafficking
R-MMU-9837999: Mitochondrial protein degradation
GO:0006351: DNA-templated transcription
R-MMU-72766: Translation
GO:0061024: membrane organization
GO:0070848: response to growth factor
GO:1903829: positive regulation of protein localization
GO:0048858: cell projection morphogenesis
PC2
PC3
