# Supplementary material for: Somatostatin-Expressing Neurons Regulate Sleep Deprivation and Recovery
Source: Genes (Basel). 2026 Jan 1;17(1):51. doi: 10.3390/genes17010051 (PMC12840664; doi:10.3390/genes17010051)
Supplement: Supplementary file 1 [file genes-17-00051-s001.zip › Supplementary File S2-Metascape.zip!/Metascape/all.tzpz10j5i/Enrichment_heatmap/HeatmapSelectedGOParent.pdf]

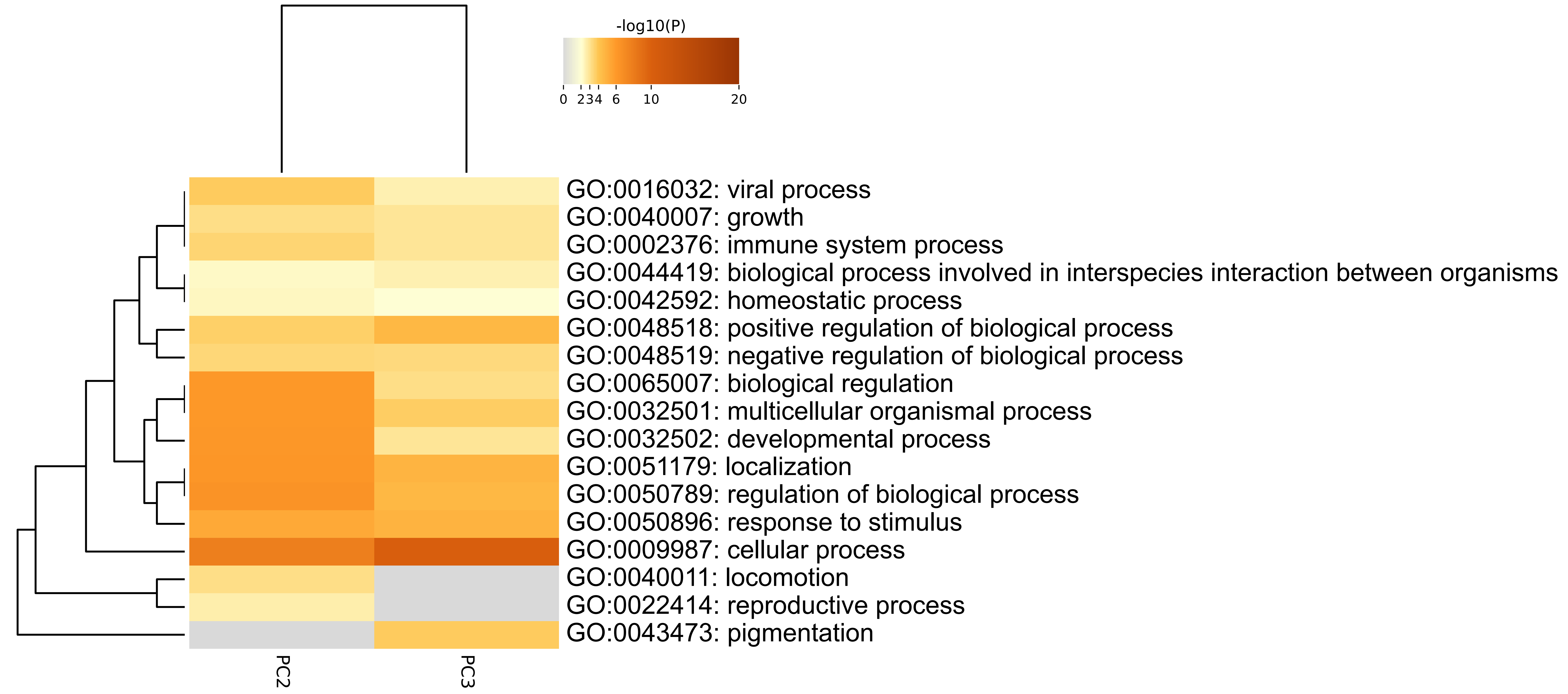
-log10(P)
0
2
3
4
6
10
20
GO:0016032: viral process
GO:0040007: growth
GO:0002376: immune system process
GO:0044419: biological process involved in interspecies interaction between organisms
GO:0042592: homeostatic process
GO:0048518: positive regulation of biological process
GO:0048519: negative regulation of biological process
GO:0065007: biological regulation
GO:0032501: multicellular organismal process
GO:0032502: developmental process
GO:0051179: localization
GO:0050789: regulation of biological process
GO:0050896: response to stimulus
GO:0009987: cellular process
GO:0040011: locomotion
GO:0022414: reproductive process
GO:0043473: pigmentation
PC2
PC3
