# Supplementary material for: Somatostatin-Expressing Neurons Regulate Sleep Deprivation and Recovery
Source: Genes (Basel). 2026 Jan 1;17(1):51. doi: 10.3390/genes17010051 (PMC12840664; doi:10.3390/genes17010051)
Supplement: Supplementary file 1 [file genes-17-00051-s001.zip › Supplementary File S2-Metascape.zip!/Metascape/all.tzpz10j5i/Enrichment_PPI/cluster_1.pdf]

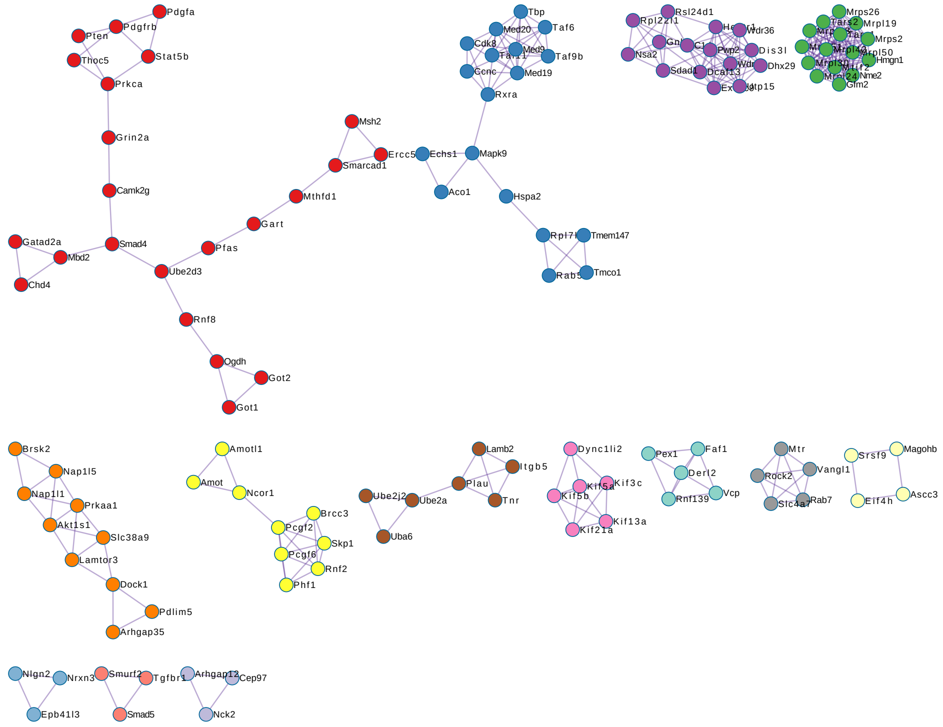
Pdgfa
Pten
Pdgfrb
Thoc5
Stat5b
Prkca
Grin2a
Camk2g
Gatad2a
Mbd2
Smad4
Chd4
Ube2d3
Pfas
Gart
Mthfd1
Smarcad1
Msh2
Ercc5
Echs1
Mapk9
Aco1
Hspa2
Rnf8
Ogdh
Got2
Got1
Tbp
Med20
Taf6
Cdk8
Med9
Taf9b
Ccnc
Med19
Rxra
Tmem147
Tmco1
Rpl22l1
Rsl24d1
Wdr36
Nsa2
Dis3l
Dhx29
Sdad1
Dcaf13
Utp15
Mrps26
Mrpl19
Mrps2
Mrpl50
Hmgn1
Nme2
Gfm2
Brsk2
Nap1l5
Nap1l1
Prkaa1
Akt1s1
Slc38a9
Lamtor3
Dock1
Pdlim5
Arhgap35
Amotl1
Amot
Ncor1
Brcc3
Pcgf2
Skp1
Pcgf6
Rnf2
Phf1
Ube2j2
Ube2a
Uba6
Lamb2
Itgb5
Plau
Tnr
Dync1li2
Kif3c
Kif5b
Kif13a
Kif21a
Pex1
Faf1
Derl2
Rnf139
Vcp
Mtr
Rock2
Vangl1
Rab7
Srsf9
Magohb
Eif4h
Ascc3
Nlgn2
Nrxn3
Epb41l3
Smurf2
Tgfbr1
Smad5
Arhgap12
Cep97
Nck2
