# Supplementary material for: Somatostatin-Expressing Neurons Regulate Sleep Deprivation and Recovery
Source: Genes (Basel). 2026 Jan 1;17(1):51. doi: 10.3390/genes17010051 (PMC12840664; doi:10.3390/genes17010051)
Supplement: Supplementary file 1 [file genes-17-00051-s001.zip › Supplementary File S2-Metascape.zip!/Metascape/all.tzpz10j5i/Enrichment_PPI/_FINAL_MCODE_ALL_PPIColorByCluster.pdf]

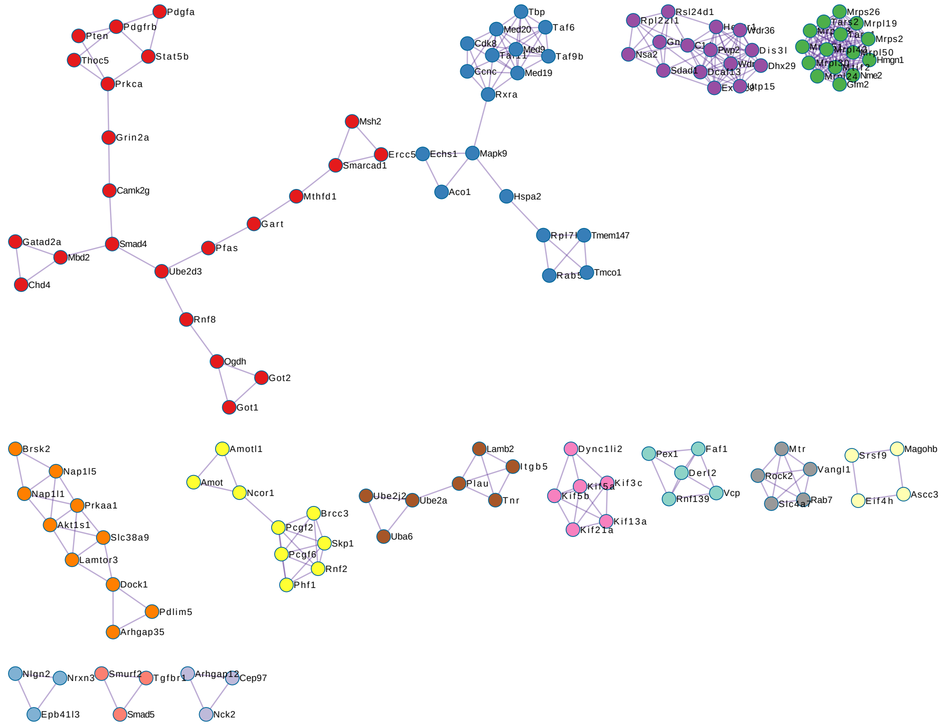

Pdgfa
Pten
Pdgfrb
Thoc5
Stat5b
Prkca
Grin2a
Camk2g
Gatad2a
Mbd2
Chd4
Smad4
Ube2d3
Pfas
Gart
Mthfd1
Smarcad1
Msh2
Ercc5
Rnf8
Ogdh
Got2
Got1
Tbp
Med20
Taf6
Cdk8
Med9
Ccnc
Med19
Taf9b
Rxra
Echs1
Mapk9
Aco1
Hspa2
Tmem147
Tmco1
Rsl24d1
Rpl22l1
Wdr36
Nsa2
Pwp2
Dis3l
Sdad1
Dcaf13
Dhx29
Utp15
Mrps26
Tars2
Mrpl19
Mrps2
Mrpl42
Mrpl50
Hmgn1
Mrpl30
Mtif2
Mrpl24
Nme2
Gfm2
Brsk2
Nap1l5
Nap1l1
Prkaa1
Akt1s1
Slc38a9
Lamtor3
Dock1
Pdlim5
Arhgap35
Amotl1
Amot
Ncor1
Brcc3
Pcgf2
Skp1
Pcgf6
Rnf2
Phf1
Ube2j2
Ube2a
Uba6
Lamb2
Itgb5
Plau
Tnr
Dync1li2
Kif3c
Kif5a
Kif5b
Kif13a
Kif21a
Pex1
Faf1
Derl2
Vcp
Rnf139
Mtr
Rock2
Vangl1
Rab7
Srsf9
Magohb
Eif4h
Ascc3
Nlgn2
Nrxn3
Epb41l3
Smurf2
Tgfbr1
Smad5
Arhgap12
Cep97
Nck2

MCODE1
MCODE2
MCODE3
MCODE4
MCODE5
MCODE6
MCODE7
MCODE8
MCODE9
MCODE10
MCODE11
MCODE12
MCODE13
MCODE14

created by
http://metascape.org
